# Ethanol consumption during gestation promotes placental alterations in IGF-1 deficient placentas

**DOI:** 10.12688/f1000research.75116.2

**Published:** 2024-03-08

**Authors:** Irene Martín-Estal, Oscar R Fajardo-Ramírez, Mario Bermúdez De León, Carolina Zertuche-Mery, Diego Rodríguez-Mendoza, Patricio Gómez-Álvarez, Marcela Galindo-Rangel, Andrea Leal López, Inma Castilla-Cortázar, Fabiola Castorena-Torres

**Affiliations:** 1 Tecnologico de Monterrey, Tecnologico de Monterrey, Monterrey, Nuevo Leon, 64710, Mexico; 2 Departamento de Biología Molecular, Centro de Investigación Biomédica del Noreste Instituto Mexicano del Seguro Social, Monterrey, Nuevo Leon, 64720, Mexico; 3 Tecnologico de Monterrey, Hospital San Jose, Monterrey, Nuevo Leon, Mexico; 4 Fundación de Investigación HM Hospitales, Madrid, Madrid, Spain

**Keywords:** placenta, IGF-1 deficiency, fetal growth restriction, ethanol.

## Abstract

**Background:**

During pregnancy, the placenta is an extremely important organ as it secretes its own hormones, *e.g.* insulin-like growth factor 1 (IGF-1), to ensure proper intrauterine fetal growth and development. Ethanol, an addictive and widely used drug, has numerous adverse effects during pregnancy, including fetal growth restriction (FGR). To date, the molecular mechanisms by which ethanol triggers its toxic effects during pregnancy, particularly in the placenta, are not entirely known. For this reason, a murine model of partial IGF-1 deficiency was used to determine ethanol alterations in placental morphology and AAH expression.

**Methods:**

Heterozygous (HZ, *Igf1 ^+/-^*) female mice were given 10% ethanol during 14 days as an acclimation period and throughout pregnancy. HZ female mice given water were used as controls. At gestational day 19, pregnant dams were sacrificed, placentas were collected and genotyped for subsequent studies.

**Results:**

IGF-1 deficiency and ethanol consumption during pregnancy altered placental morphology, and decreased placental efficiency and aspartyl/asparaginyl β-hydroxylase (AAH) expression in placentas from all genotypes. No differences were found in *Igf1*, *Igf2*, *Igf1r* and *Igf2r* mRNA expression in placentas from all groups.

**Conclusions:**

IGF-1 deficiency and ethanol consumption throughout gestation altered placental development, suggesting the crucial role of IGF-1 in the establishment of an adequate intrauterine environment that allows fetal growth. However, more studies are needed to study the precise mechanism to stablish the relation between both insults.

## Abbreviations

AAH: aspartyl/asparaginyl β-hydroxylase

AKT: protein kinase B

cDNA: complementary DNA

DAB: diaminobenzidine

EDTA: ethylenediaminetetraacetic acid

ELISA: enzyme-linked immunosorbent assay

eNOS: endothelial nitric oxide synthase

FASD: fetal alcohol spectrum disorders

FGR: fetal growth restriction

HRP: horseradish peroxidase

HZ: heterozygous

IGF-1: insulin-like growth factor 1

IGF-2: insulin-like growth factor 2

IGFBP-1: IGF-1 binding protein 1

IGFBP-3: IGF-1 binding protein 3

IGF1R: IGF-1 receptor

IGF2R: IGF-2 receptor

INSR: insulin receptor

KO: knock-out

MAPK: mitogen-activated protein kinase

PBS: phosphate buffered saline

PCR: polymerase chain reaction

PI3K: phosphoinositide-3-kinase

RT-qPCR: reverse transcription coupled to polymerase chain reaction

SD: standard deviation

WT: wild type

## 1. Introduction

Throughout pregnancy, the placenta is an essential organ for both mother and fetus; being the major determinant of intrauterine growth and serving as a protective barrier against external and internal insults. ^1^

Insulin-like growth factor 1 (IGF-1) is a pleiotropic hormone with several functions: mitochondrial protection, ^2^ cell proliferation and survival, ^3^ tissular growth and development. ^4^ It regulates placental morphogenesis and hormone secretion into umbilical and maternal circulations, processes that are indispensable for fetal development. ^5^ Furthermore, IGF-1, as well as insulin-like growth factor 2 (IGF-2), are key providers of placental resource allocation either for development or for response to external and environmental insults. ^5^^–^^7^

To this day, numerous IGF-1 deficiency conditions have been described in humans, *e.g.* fetal growth restriction (FGR). ^8^ FGR is a disorder where reduced levels in both IGF-1 and IGF-1 binding protein 3 (IGFBP-3) are observed, suggesting that reduced IGF-1 concentration in the fetus, mother and/or placenta may contribute to growth restriction. ^8^^,^^9^ Also, FGR, among other harmful consequences, such as fetal death, miscarriage, low birth weight, premature birth and fetal alcohol spectrum disorders (FASD), can be a result of ethanol consumption during pregnancy . ^10^^,^^11^

Current experimental studies have shown that chronic exposure to high levels of ethanol during pregnancy reduces fetal weight ^12^ as well as maternal and fetal plasmatic levels of both IGF-1 and IGFBP-3. ^13^ Also, ethanol increases both IGF-1 binding protein 1 (IGFBP-1, an inhibitory protein for IGF-1) and IGF-2 levels, ^13^ inhibits insulin, IGF-1 and IGF-2 placental gene expression and/or secretion ^12^^,^^14^ and reduces activities of both insulin and IGF-1 receptors ^15^^,^^16^; thus, altering IGF-1 bioavailability and its downstream signaling.

The molecular mechanism for ethanol toxic effects during pregnancy, specially in the placenta, it is not totally understood. Several studies in animal models of ethanol consumption suggest that this molecule alters the insulin and IGF-1 signaling pathway, impairing cell viability, metabolism, homeostasis, and hence, normal placental growth and development. ^9^^,^^14^^,^^17^

Aspartyl-(asparaginyl) β-hydroxylase (AAH), a type 2 transmembrane protein that hydroxylates epidermal growth factor-like domains of proteins that have a functional role in cell motility and invasion, is regulated by the IGF-1 signaling pathway. ^18^ AAH is overexpressed in trophoblast cells, which are motile and invasive epithelial cells that mediate the appropriate development of placenta and implantation. ^19^ Experimental studies in murine models with chronic ethanol exposure, showed a reduced expression of IGF-1 in placentas, being associated with decreased expression of AAH in trophoblastic cells, suggesting a role of the IGF-1 signaling pathway in placentation and fetal development. ^9^^,^^14^

For this reason, as either IGF-1 deficiency or ethanol consumption during pregnancy produces FGR, the aim of the present study was to determine whether IGF-1 partial deficiency is responsible for placental alterations in morphology and AAH expression, as well as if chronic ethanol exposure during gestation contributed to these placental changes.

## 2. Methods

### 2.1 Animals and experimental design

As previously reported, IGF-1 heterozygous mice were obtained by cross-breeding transgenic mice, line 129SV and Igf1 ^tm1Ts^/ImJ (003258, Jackson Laboratory, Maine, USA) and CD1 (non-consanguineous, Circulo A.D.N S. A de C.V., Ciudad de Mexico, Mexico). ^20^

Animals were housed in cages in a room with 12-hour light/dark cycle, constant humidity (50–55%) and temperature (20–22°C). Food (PicoLab ^®^ Rodent Diet 20 5053*, Missouri, USA) and water were given *ad libitum.* All experimental procedures were performed in compliance with the National Institutes of Health Guide for the Care and Use of Laboratory Animals (NIH Publications No. 8023, revised 1978), the NORMA Oficial Mexicana (NOM-062-ZOO-1999) for technical specifications to produce, care and use of laboratory animals and the ARRIVE guidelines. Also, all experimental procedures were approved by the Institutional Committee for the Care and Use of Laboratory Animals of Tecnologico de Monterrey (Protocol 2018-006). All efforts were made to ameliorate any suffering of animals, such as minimal handling, adequate space for living and no other experimentation or pain inducing procedures.

The sample size was calculated using the resource equation method, ^21^ where the value of E is calculated and should lie between 10 and 20 to be considered adequate: E = Total number of animals − Total number of groups = (7 × 2) − 2 = 12.

Two randomly selected experimental groups of heterozygous (HZ, *Igf1 ^+/−^*) female mice 16±8 weeks old were randomly included in the experimental protocol: one control group given water (HZ–Control, *n* = 7), and other group given food grade ethanol (HZ–Ethanol, *n* = 7) (purity: 96%; La Fe, Nuevo Leon, Mexico).

**Figure 1.**
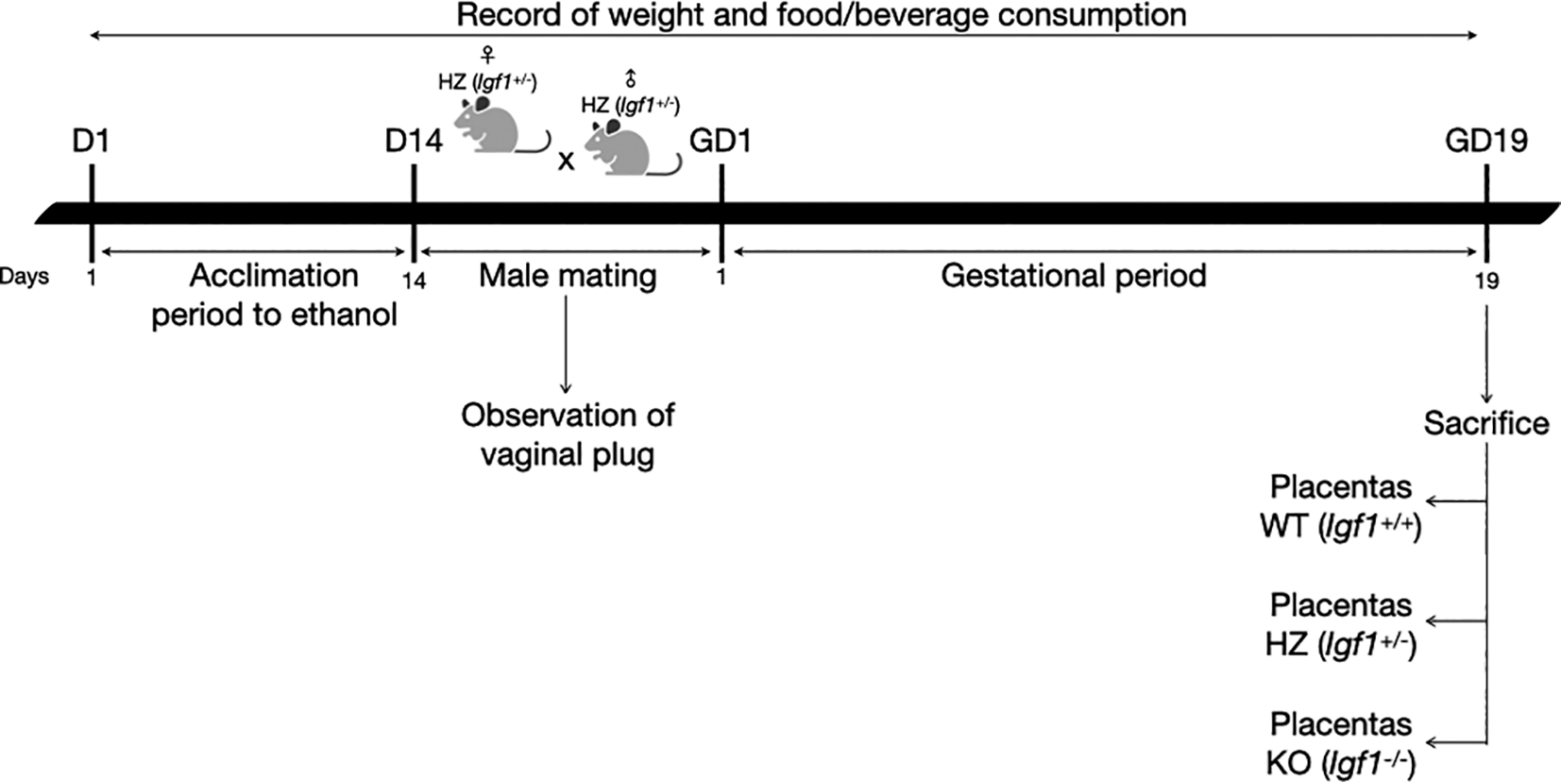
Diagram of the experimental procedure. D: day; GD: gestational day.

An adaptation period to ethanol (14 days) was performed, where HZ female mice were given increasing concentrations of 2%, 5% and 10% of ethanol in water, each introduced after a 48-hour acclimation to the previous concentration, according to Kleiber *et al.* ^22^ During this period, control HZ female mice were provided water ( Figure 1).

After the acclimation period to ethanol, HZ male mice were mated overnight with HZ female mice. To avoid ethanol consumption from males during mating, water was given to the ethanol group. Once the vaginal plug was observed, it was considered gestational day one and males were removed. Throughout gestation, 10% ethanol or water was given to pregnant dams (HZ-Ethanol and HZ-Control; respectively), food and beverage consumption and weight gain were monitored throughout the experimental protocol. Nonpregnant females from HZ-Ethanol group were given 10% ethanol during 24 hours and then mated again with male mice. If vaginal plug was not observed after this second mating, ethanol exposed female mice were sacrificed by cervical dislocation. On the other hand, nonpregnant females from HZ-Control group were mated again until the presence of the vaginal plug was observed.

At gestational day 19 (before the end of the gestational period in mice), pregnant females were sacrificed by cervical dislocation. ^23^^,^^24^ Subsequently, blood was collected by cardiac puncture and a caesarean section was performed to obtain fetuses and placentas, which were measured and weighted ( Figure 1).

Tails from fetuses were cut for genotype determinations. Fetuses and placentas were stored randomly blind in either paraformaldehyde 4%, for histology and immunohistochemistry analyses, or liquid nitrogen, for reverse transcription coupled to polymerase chain reaction (RT-qPCR) determinations.

### 2.2 Genotyping of animals

DNA was extracted from tails of fetuses using the Wizard Genomic DNA Purification Kit (A1125, Promega, Wisconsin, USA) following manufacturer’s instructions, and stored at −20°C until analysis. End-point polymerase chain reaction (PCR) (Veriti 96 well Thermal Cycler, Applied Biosystems, California, USA) was carried out for genotyping using the following set of primers for *Igf1* gene: WT forward 5′-TTCATGCCACACTGCTCTTC-3′; common 5′-AGAGGGGATGGGAGAGCTAC-3′; and mutant forward 5′-GCCAGAGGCCACTTGTGTAG-3′. All primers were acquired from IDT (Iowa, USA). Secondly, conventional PCR analysis was achieved using the GoTaq Green Master Mix (M712C, Promega, Wisconsin, USA) following manufacturer’s instructions.

### 2.3 Serum IGF-1 circulating levels at gestational day 19

In order to evaluate serum IGF-1 circulating levels in HZ dams at gestational day 19, sera from control (WT) mice treated both with water ( *n* = 5) or ethanol ( *n* = 5) were also used. Serum IGF-1 levels were determined by enzyme-linked immunosorbent assay (ELISA) using the Mouse/Rat IGF-1 commercial kit (22-IG1MS-E01, Alpco, New Hampshire, USA), following manufacturer’s instructions. The signal was measured using a spectrophotometer Synergy HT (Biotek, Vermont, USA) and data were interpreted using Gen5 Data Analysis Software (Biotek, Vermont, USA).

### 2.4 Placental histological study

For histopathological analysis, placental samples from all experimental groups were fixed in 4% paraformaldehyde diluted in 10 mM phosphate buffered saline (PBS) solution for 24 hours. Once samples were properly fixed, they were dehydrated in increasing ethanol concentrations and were embedded in liquid paraffin using the automated equipment Leica TP 1020 (Leica, Wetzlar, Germany). Sections 4 μm thick were cut using a Reichert Jung Leica BC2030 Histocut Rotary Microtome (Leica, Hesse, Germany) and subsequently stained with hematoxylin-eosin (HHS32 and 2853, Sigma-Aldrich, Missouri, USA). Finally, all histological preparations were evaluated independently by three observers (double-blind) in a Zeiss Axio Imager M2 microscope (Zeiss, Baden-Württemberg, Germany) to establish morphology alterations in the placenta due to ethanol consumption throughout gestation.

All images were analysed using the processing package ImageJ (National Institutes of Health, Maryland, USA) (RRID:SCR_003070). The area of the junctional zone of the placenta was evaluated according to the presence of trophoblasts in this placental zone. Three images were taken per sample of at least three individuals from each group and genotype.

### 2.5 Immunohistochemical staining

Paraffin-embedded placental tissues were deparaffinized and rehydrated in decreasing ethanol concentrations. Sample sections were incubated with 3% hydrogen peroxide at room temperature in darkness for 30 minutes to inactivate endogenous peroxidases. Retrieval of antigen was induced with 2 mM ethylenediaminetetraacetic acid (EDTA) pH 8.0 and 50 mM Tris–HCl pH 9.0 by microwave heating at 100°C for 8 minutes. Sections were incubated overnight at 4°C with specific primary antibodies: rabbit anti-ASPH (362200, USBiological Life Sciences, Massachusetts, USA) diluted at 1:200; or rabbit anti-β actin (ab8227, Abcam, Cambridgeshire, United Kingdom) diluted at 1:1,000, the latter being an endogenous control. Afterwards, primary antibodies sections were incubated with goat anti-rabbit IgG H&L (horseradish peroxidase, HRP) (ab205718, Abcam, Cambridgeshire, United Kingdom) diluted at 1:5,000 for one hour at room temperature. Staining was developed using Steady Diaminobenzidine (DAB)/Plus kit (ab103723, Abcam, Cambridgeshire, United Kingdom) following manufacturer’s instructions. Negative controls were conducted by omitting the primary antibody.

Digital images of tissue sections (five images per placenta) were captured by three observers (double-blind) using a Zeiss Axio Imager M2 microscope. All images were analysed using the processing package ImageJ (National Institutes of Health, Maryland, USA).

### 2.6 Gene expression studies in placentas via RT-qPCR

RNA was isolated from placentas using Trizol reagent (15596026, Invitrogen, California, USA). Quality was checked by the A _260_/A _280_ ratio using the Nanodrop 2000 Spectrophotometer (Thermo Scientific, Massachusetts, USA), whereas integrity was determined by electrophoresis in 1% agarose gel. Samples were stored at −80°C until analysis. Subsequently, the SuperScript III First-Strand Synthesis System (Invitrogen, California, USA) was utilized to prepare complementary DNA (cDNA) from 500 ng of the total RNA, according to the manufacturer’s instructions.

For the PCR analyses, TaqMan Universal PCR Master Mix (Thermofisher, Massachusetts, USA) was used in a total volume of 20 μL containing 400 nM of each oligonucleotide and 200 mM of specific Taqman ^®^ probes for particular genes, supplied by Applied Biosystems (California, USA) (Supplementary table 1). The assays were performed in a 96-well reaction plate on a Quant Studio 3.0 thermocycler (Applied Biosystems, California, USA). Ribosomal 18S RNA expression was used as an endogenous control (Applied Biosystems, California, USA).

Each sample was run in triplicate, and negative controls were included in the same plate. Results were analyzed using the comparative threshold cycle method for relative gene expression. ^25^

### 2.7 Statistical analysis

All data are represented by mean±standard deviation (SD). Significance was estimated with non parametric (Mann–Whitney U test and Kruskal–Wallis test) or parametric (Student T test and ANOVA) statistical tests, using Mann–Whitney U test or Student T test to compare between two groups (for example between HZ-Control and HZ-Ethanol groups), whereas comparisons among more than two groups were carried out using the Kruskal-Wallis test or ANOVA (for example, comparisons between WT, HZ and knock-out (KO) placentas). To evaluate the effect of the two independent variables in the present study (ethanol consumption and IGF-1 deficiency) a general linear model was performed. Differences were considered significant at a level of *p* < 0.05. Statistical analyses were performed on SPSS 26 (IBM, New York, USA) (RRID:SCR_019096) and graphs were generated on Prism 8.2.1 (GraphPad Software, California, USA) (RRID:SCR_002798).

## 3. Results

### 3.1 IGF-1 serum levels in pregnant dams chronically exposed to ethanol at gestational day 19

In order to validate IGF-1 serum levels in the present experimental model, control (WT) pregnant female mice treated with water or ethanol were used. At gestational day 19, as expected, IGF-1 serum levels in HZ pregnant dams (HZ-Control, 454 ± 58 ng/mL, and HZ-Ethanol, 422 ± 43 ng/mL) were decreased compared to WT female mice (WT-Control, 739 ± 48 ng/mL, and WT-Ethanol, 795 ± 147 ng/mL) ( *p* < 0.001). HZ-Control and HZ-Ethanol dams had significant lower IGF-1 serum levels than WT-Control female mice ( *p* < 0.05 and *p* < 0.01, respectively). Interestingly, a possible synergic effect, that decreased IGF-1 serum levels, was observed by the partial IGF-1 deficiency and ethanol consumption (HZ-Ethanol) during gestation compared to WT-Control group ( *p* < 0.01).

### 3.2 Alterations in placental morphology due to ethanol consumption during gestation

**Figure 2.**
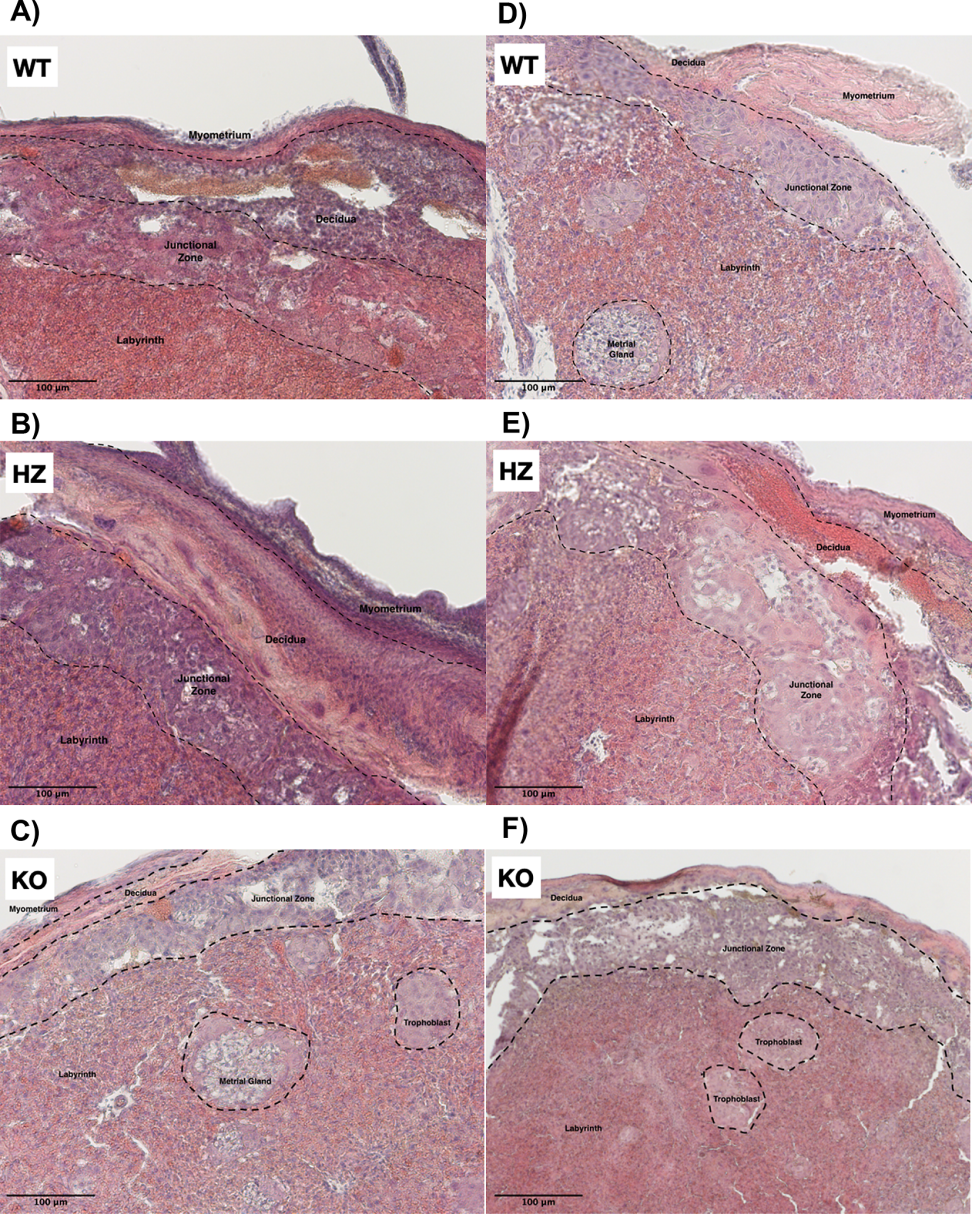
Effect of gestational ethanol consumption on placental morphology on gestational day 19. Placentas from WT, HZ and KO fetuses from pregnant dams given water (A, *n* = 5; B, *n* = 6; and C, *n* = 6) or ethanol (D, *n* = 7; E, *n* = 6; and F, *n* = 5) were stained with hematoxylin & eosin and examined by light microscopy. Representative images from one placenta of fetuses from each group are included. Black arrows indicate trophoblast islets, suggesting an abnormal migration. IGF-1 deficiency promoted placental disorganization, especially in the junctional zone; ethanol consumption during gestation exacerbated such disorganization. Original magnification 100×. Scale bar = 100 μm.

The placenta is conformed of several strata, such as myometrium, decidua, junctional zone and labyrinth (Extended data: figure 1). In the WT placentas from the control group all morphology and structure that characterizes these layers were well defined ( Figure 2A). It was observed that IGF-1 deficiency promoted a disorganization in placental strata, specially in the junctional zone, where trophoblast cells were observed ( Figure 2B). Remarkably, an abnormal placental development was observed in KO placentas from the control group, where trophoblasts were arrested in islets ( Figure 2C). Ethanol consumption throughout gestation exacerbated placental layer disorganization and promoted throphoblast arrest in islets in all groups (WT, HZ and KO) ( Figures 2D, 2E and 2F). Additionally, ethanol use altered trophoblast migration ( Figure 2D) and promoted placental hypoplasia, particularly in HZ and KO placentas ( Figures 2E and 2F). Due to the variability between all experimental groups, no significant differences were observed in the area of the junctional zone of the placenta (data not shown); in this area a clear disorganization is detected with both IGF-1 deficiency and ethanol consumption.

### 3.3 Impairments in placental efficiency due to ethanol consumption and IGF-1 deficiency

**Figure 3.**
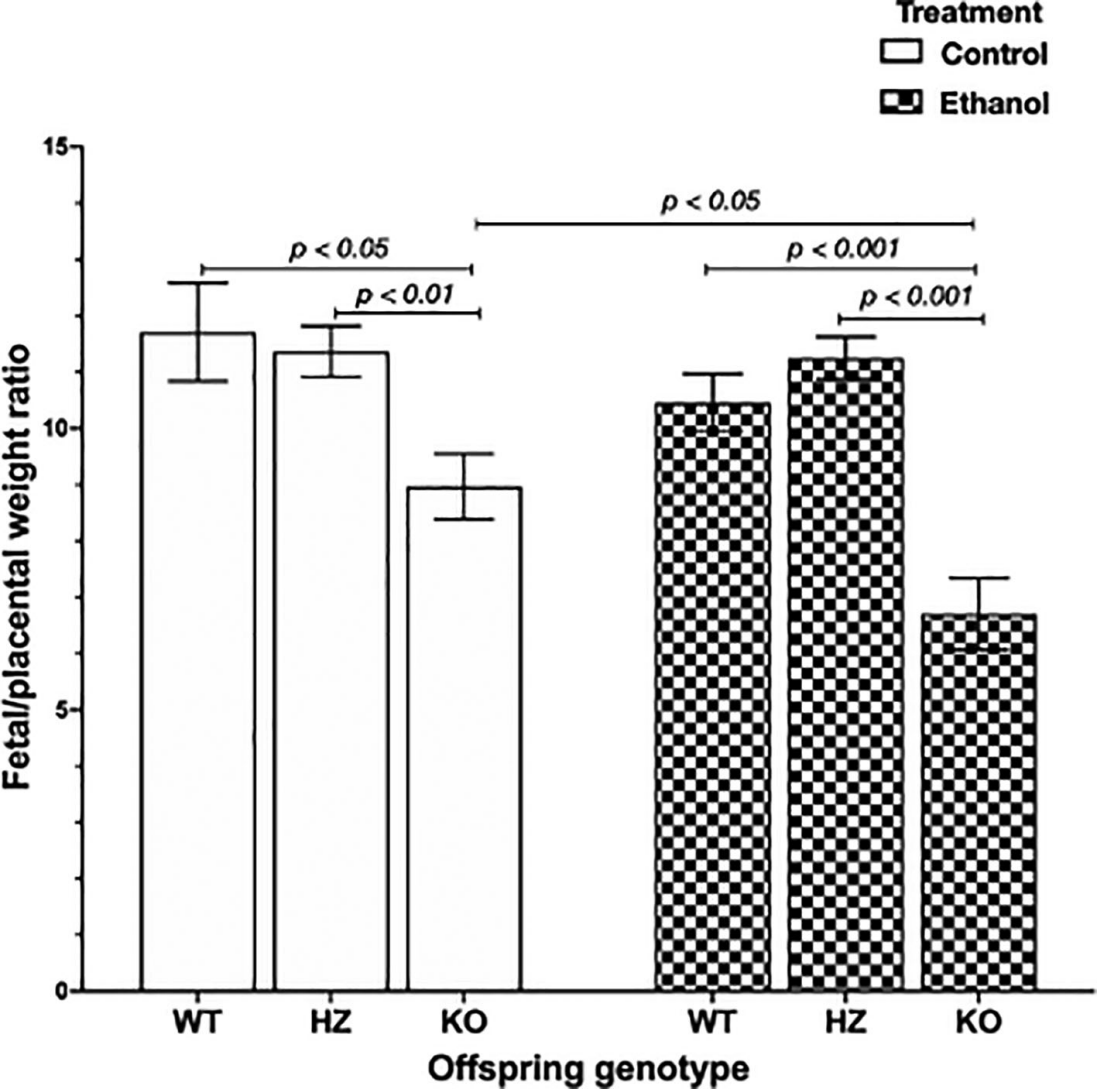
Placental efficiency represented as feto/placental weight ratio in all experimental groups (Control: WT, *n* = 9; HZ, *n* = 32; KO, *n* = 22; Ethanol: WT, *n* = 23; HZ, *n* = 44; KO, *n* = 16). Data are represented as mean ± standard deviation. The statistical tests used were Student T test to compare between two groups, and general linear model to evaluate the effect of the two independent variables in the present study (ethanol consumption and IGF-1 deficiency). Differences were considered significant at a level of *p* < 0.05.

Placental efficiency is frequently defined as the grams of fetus produced per gram of placenta, being an indicator of the ability of this organ to maintain an adequate nutrient supply to the fetus. ^26^^–^^28^ The chronic exposure to ethanol during gestation resulted in a significant reduction in placental efficiency ( *p* < 0.001) compared to control groups. Subsequently, genotype offspring outcome was evaluated, showing that IGF-1 deficiency significantly decreased placental efficiency in KO placentas compared to WT placentas ( *p* < 0.05) and HZ placentas ( *p* < 0.01) from control group ( Figure 3). Also, ethanol consumption during gestation significantly reduced placental efficiency in KO placentas compared to WT and HZ placentas from ethanol treated group ( *p* < 0.001 in both cases) ( Figure 3). Markedly, there was a significant diminution in 0.75-fold in placental efficiency in KO ethanol treated placentas compared to KO placentas from control group ( *p* < 0.05) ( Figure 3).

### 3.4 Immunohistochemical analysis of AAH placental expression

**Figure 4.**
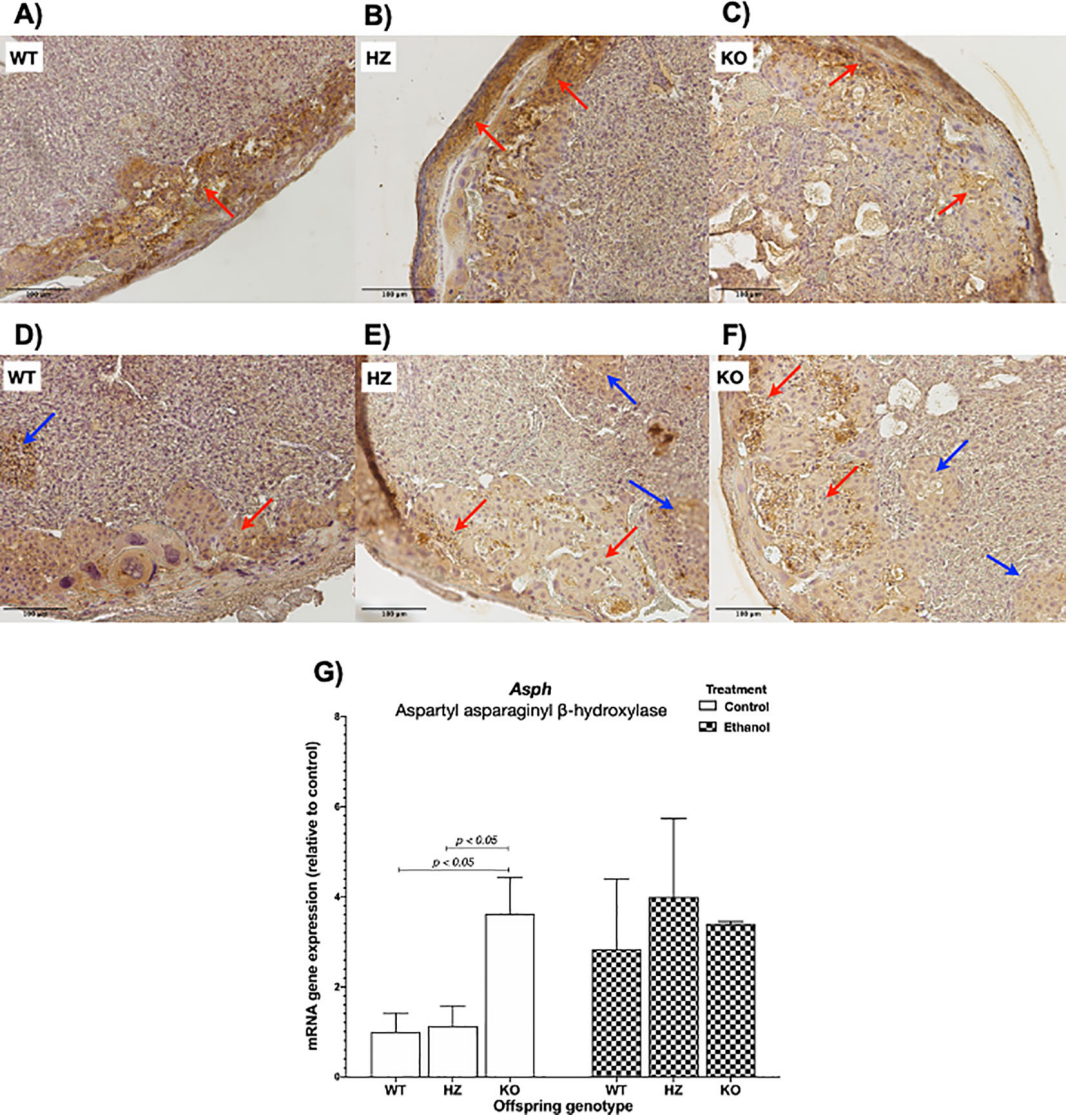
Effect of ethanol consumption during gestation in AAH levels analyzed by immunohistochemistry in placentas from WT, HZ and KO mice given water (A, *n* = 5; B, *n* = 6; anc C, *n* = 6) or ethanol (D, *n* = 7; E, *n* = 6; and F, *n* = 5). Original magnification 100x. Representative images from one mice of each group are included. Red arrows denote AAH expression in the junctional zone of the placenta, where trophoblast cells are expressed. Blue arrows indicate trophoblast islets. G) Effect of ethanol consumption during gestation in *Asph* gene expression in placentas from WT, HZ and KO fetuses from pregnant dams given water or ethanol ( *n* = 4 per group). The statistical tests used were Mann-Whitney U to compare between two groups, ANOVA or Kruskal-Wallis test to compare between more than two groups, and general linear model to evaluate the effect of the two independent variables in the present study (ethanol consumption and IGF-1 deficiency). Differences were considered significant at a level of *p* < 0.05.

To determine ethanol’s harmful effects on placental development, protein expression levels of AAH were determined by immunohistochemistry. AAH was predominantly expressed in the junctional zone, as shown in WT placentas from control group ( Figure 4A). IGF-1 deficiency revealed a decrease in AAH expression within the junctional zone in both HZ and KO placentas compared with WT placentas from control group ( Figures 4B and 4C). Ethanol consumption during gestation also decreased AAH expression, especially in HZ and KO placentas, denoting the aforementioned alterations in placental morphology, particularly in the junctional zone, where trophoblasts are arrested in islets, avoiding their correct migration towards the junctional zone for an appropriate placental development ( Figures 4D, 4E and 4F).

### 3.5 The effect of ethanol consumption during gestation in placental expression of the IGF-1 signaling pathway

The placental expression of components from the IGF-1 signaling pathway (Insulin-like growth factor 1 ( *Igf1*), Insulin-like growth factor 2 ( *Igf2*), Insulin-like growth factor 1 receptor ( *Igf1r*), Insulin-like growth factor 2 receptor ( *Igf2r*) and Aspartyl/asparaginyl β-hydroxylase ( *Asph*)) was analyzed by RT-qPCR. No significant differences were found in *Igf1* and *Igf2* expression level between both treatments (control and ethanol). When grouped by genotype, no significant differences were found ( Table 1).

**Table 1.** Reverse transcription coupled to polymerase chain reaction analyses revealing gene expression of Insulin-like growth factor 1 signaling pathway related proteins.

Gene description	Gene name	Control			Ethanol		
		WT Fold change (SD)	HZ Fold change (SD)	KO Fold change (SD)	WT Fold change (SD)	HZ Fold change (SD)	KO Fold change (SD)
Insulin-like growth factor 1	*Igf1*	1.00 (0.75)	0.70 (0.52)	2.34 (2.23)	0.38 (0.21)	0.92 (1.40)	0.66 (0.56)
Insulin-like growth factor 2	*Igf2*	1.00 (2.02)	2.11 (3.99)	0.79 (1.98)	1.87 (4.03)	1.49 (2.32)	2.39 (3.69)
Insulin-like growth factor 1 receptor	*Igf1r*	1.00 (0.56)	1.45 (0.55)	3.51 (1.52)	5.44 (3.45)	3.52 (0.77)	5.71 (1.86)
Insulin-like growth factor 2 receptor	*Igf2r*	1.00 (0.38)	0.95 (0.25)	1.57 (0.46)	1.79 (0.43)	1.88 (0.59)	1.82 (0.40)
Aspartyl/asparaginyl β-hydroxylase	*Asph*	1.00 (0.42)	1.13 (0.45)	3.62 (0.82) ^a^^,^^b^	2.84 (1.57)	4.00 (1.74)	3.40 (0.05)

The statistical tests used were Mann-Whitney U or Student T test to compare between two groups, and general linear model to evaluate the effect of the two independent variables in the present study (ethanol consumption and IGF-1 deficiency). Differences were considered significant at a level of *p* < 0.05. The letters showed the significant differences founded: ^a^ *p* < 0.05 KO *vs* WT; ^b^ *p* < 0.05 KO *vs* HZ.

Regarding *Igf1r* and *Igf2r* expression, a significant increase in *Igf1r* was observed with ethanol consumption throughout gestation ( *p* < 0.05). When grouped by genotype, no significant differences were observed ( Table 1).

When *Asph* expression levels were evaluated, no significant differences were shown between both treatments (water or control, and ethanol). When grouped by genotype, a significant increase in *Asph* expression was observed in KO placentas compared to WT ( *p* < 0.05) and HZ ( *p* < 0.05) placentas from control group, whereas ethanol treatment seemed to have an increase effect in WT and HZ placentas ( Figure 4G).

## 4. Discussion

The placenta is an exciting organ, as it performs crucial roles during intrauterine development: nutrient and oxygen supply to the fetus, exchange of waste products between mother and fetus, metabolism of various molecules, and hormone production and secretion. Also, this organ can operate as a barrier to avoid infections, maternal diseases and propagation of xenobiotics, contributing to a suitable fetal development. ^29^ However, the placenta continues to be an mysterious organ, as there are still many questions to be solved.

Gestation is an intricate process where, additionally to the reorganization of the mother’s organs, numerous adaptations in the endocrinological axis occur to prepare fetal development. For example, IGF-1 serum levels increase during pregnancy, especially in the first trimester, and continue to rise throughout this period ^30^ in order to provide a suitable intrauterine environment for fetal and placental growth. ^31^ Results in this project showed that ethanol consumption during gestation significantly affects IGF-1 serum levels in HZ pregnant dams, according to existing literature. ^32^

The placenta is composed of by several strata, such as myometrium, decidua, junctional zone (cytotrophoblast and syncytiotrophoblast layers) and labyrinth, each presenting certain characteristics that allow the placenta to achieve its functions properly. ^33^ The major source of placental hormones is the syncytiotrophoblast layer, *e.g.,* IGF-1 is produced and secreted in this stratum. ^34^^,^^35^ Recently, reduced IGF-1 levels have been associated with several pathologies, such as FGR, ^8^^,^^36^^,^^37^ a consequence of ethanol consumption. ^38^ Results herein revealed that both IGF-1 deficiency and ethanol consumption during gestation promoted placental disorganization, particularly in the junctional zone, where trophoblasts are arrested in islets, highlighting the crucial role of IGF-1 in placental development. It is important to notice that also IGF-2 has an important role in fetal growth due to its direct anabolic actions within the fetus, and by its modulation of placental supply capacity, which may be a compensatory mechanism for IGF-1 deficiency. ^39^

According to these results, ethanol consumption during gestation in the present experimental model aggravated placental disorganization and promoted placental hypoplasia, particularly in IGF-1 deficient placentas, suggesting a synergic effect between both issues, that could lead to poor placental development and, hence, an abnormal fetal growth. One of the main determinants of placental formation and function are trophoblasts, located in junctional and labyrinth zones of the placenta, the major cell type affected by ethanol consumption. Ethanol consumption during gestation can lead to an altered trophoblast motility and invasion, resulting in a distorted placental barrier thickness and abnormal development that can promote an aberrant nutrient exchange, producing impairments in fetal growth and development. ^40^^,^^41^

Several studies conducted in mice, pigs and sheeps have disclosed that IGF-1 exogenous treatment during gestation improves nutrient transport and waste exchange between mother and fetus, enhancing fetal growth and development. ^26^ In this context, lower placental efficiency results in reduced birth weight, which translates as an inefficient placenta that failed to adapt its nutrient supply to meet the demands of the rapidly growing fetus. ^27^^,^^28^ Results herein showed that IGF-1 deficiency decreased placental efficiency especially in KO placentas, being these fetuses the smallest ones, highlighting the crucial role of this hormone in placental development. ^42^

Results herein reported showed that ethanol decreased placental efficiency specially in KO placentas, suggesting a key role of IGF-1 in placental formation and function necessary for a suitable fetal development. In this sense, ethanol consumption during gestation also reduces placental efficiency due to reductions in angiogenesis-related proteins. ^43^^,^^44^ However, the results suggest that ethanol consumption during gestation did not gravely affect the development of HZ embryo-placenta units. This could be due to the intricate genetics of the placenta, being noted that it is an organ conformed of a fetal part and a maternal part, which are in constant communication. Both parts are necessary for the appropriate development of this organ, being able to be affected in the same way by external factors, such as IGF-1 deficiency and/or ethanol consumption.

*In vivo* experimental studies of ethanol consumption during gestation have revealed that ethanol use diminishes AAH expression (both mRNA and protein levels) and, hence, trophoblast survival, motility and invasion; resulting in distorted vascular remodeling and placentation. ^14^^,^^45^^,^^46^ *In vitro* studies with human trophoblastic cells have revealed that small interfering RNA inhibition of AAH reduced Notch signaling, impairing trophoblastic cell motility, both effects related to altered fetal growth. ^47^ Our results showed a decrease in AAH protein expression due to IGF-1 deficiency, mainly in the junctional zone, where trophoblast cells are located. Ethanol consumption throughout gestation promoted the arrest of trophoblasts in islets and also reduced AAH expression in the junctional zone of HZ and KO placentas, thus altering placental morphology. Regarding to mRNA expression levels, IGF-1 deficiency showed a significant increase in *Asph* expression in KO placentas from control group, while ethanol consumption during gestation also exhibited an increase in *Asph* in WT and HZ placentas, suggesting that a mechanism is taking place in order to promote IGF-1 downstream signaling expression to try to stimulate placental development despite IGF-1 deficiency and ethanol consumption. Also of note, although there is no information on other proteins that regulate AAH in the placenta, there are other protein entities that modulate its expression and would be interesting to evaluate. ^48^

IGF-1 plays a crucial role in placental and fetal development through binding to its putative receptor (IGF1R). Also, IGF-1 can bind to IGF-2 receptor (IGF2R) or to insulin receptor (INSR), but with lower affinity. The majority of these receptors are members of the tyrosine kinase family, activating two main downstream signaling pathways: the mitogen-activated protein kinase (MAPK) and the phosphoinositide-3-kinase (PI3K)/protein kinase B (AKT) cascades, both related to cell survival, growth and proliferation, *e.g.* trophoblast cell migration to establish the maternal–fetal interface that allows nutrient exchange between mother and fetus across the placenta. ^35^ Several studies conducted in human FGR placentas have shown an increase in *Igf2* and *Igf1r* expression levels and activated INSR, and a reduction in IGF1R protein content, with no change in *Igf1*, *Insr* and *Igf2r* expression levels, suggesting a placental compensatory mechanism in response to growth retardation. ^49^^,^^50^ In contrast, experimental studies in rats have shown reduced *Igf1r* expression in FGR placentas, with no change in *Insr* expression, suggesting a decreased nutrient delivery across the placenta that would reduce the growth-promoting effects of insulin-like growth factors. ^51^ In this way results herein disclosed that no significant differences were observed in *Igf1*, *Igf2*, *Igf1r* and *Igf2r* placental expression levels due to IGF-1 deficiency. These differing results suggest that more studies are needed to elucidate the role of IGF-1 downstream molecules in intrauterine growth.

The present study had some limitations. RT-qPCR analyses have revealed that the regulation of the IGF-1 signaling pathway in the placenta is very complex, especially when there are two independent variables (IGF-1 deficiency and ethanol consumption) to analyze. More studies are needed to investigate the regulation of this cascade at protein level, such as the phosphorylation level of the molecular components of the IGF-1 signaling pathway or caspase levels to observe apoptosis. Additionaly, mouse placentas have a small size, so it would be more convenient to use rat placentas, which are even more similar to human placentas, allowing a better separation and analysis of placental layers. The present study showed that IGF-1 deficiency and ethanol consumption during pregnancy impaired AAH expression in the placenta. However, more studies are needed to determine the crucial role of this enzyme in trophoblastic cell migration to promote placentation. These future experiments would allow detailed study the placenta as a valuable organ to serve as a diagnostic tool to identify novel biomarkers for detecting the outcome of IGF-1 deficiency and/or ethanol’s teratogenicity.

## 5. Conclusions

To conclude, the present study discloses that both IGF-1 deficiency and ethanol consumption during pregnancy promote placental disorganization, particularly in the junctional zone where trophoblast cells are expressed, and decrease placental efficiency, thus altering placental development and fetal growth. In addition, both parameters alter AAH protein expression in the placenta, suggesting an abnormal placental development.

## Data Availability

Zenodo: Ethanol consumption during gestation promotes placental alterations in IGF-1 deficient mice. https://doi.org/10.5281/zenodo.5750155 ^52^ This project contains the following underlying data:
-
Figure 2A_H&E Image Placenta WT (Control) 10×.tif -
Figure 2B_H&E Image Placenta HZ (Control) 10×.tif -
Figure 2C_H&E Image Placenta KO (Control) 10×.tif -
Figure 2D_H&E Image Placenta WT (Ethanol) 10×.tif -
Figure 2E_H&E Image Placenta HZ (Ethanol) 10×.tif -
Figure 2F_H&E Image Placenta KO (Ethanol) 10×.tif -
Figure 4A_AAH 1/200 Image Placenta WT (Control) 10×.tif -
Figure 4B_AAH 1/200 Image Placenta HZ (Control) 10×.tif -
Figure 4C_AAH 1/200 Image Placenta KO (Control) 10×.tif -
Figure 4D_AAH 1/200 Image Placenta WT (Ethanol) 10×.tif -
Figure 4E_AAH 1/200 Image Placenta HZ (Ethanol) 10×.tif -
Figure 4F_AAH 1/200 Image Placenta KO (Ethanol) 10×.tif -
Figure 3_Placental efficiency Raw data.xlsx -
Table 1 and Figure 4G_RT-PCR Raw data.xlsx - Raw data for RT-qPCR analysis.xls Zenodo: Ethanol consumption during gestation promotes placental alterations in IGF-1 deficient mice. https://doi.org/10.5281/zenodo.5750155 ^52^ This project contains the following extended data:
- Extended data Figure 1_H&E Image Placenta 1.25×.tif Zenodo: Ethanol consumption during gestation promotes placental alterations in IGF-1 deficient mice. https://doi.org/10.5281/zenodo.5750155 ^52^
- ARRIVE checklist.pdf (The ARRIVE guidelines 2.0) Data are available under the terms of the Creative Commons Attribution 4.0 International license (CC-BY 4.0).
