# Supplementary material for: Ethanol consumption during gestation promotes placental alterations in IGF-1 deficient mouse placentas
Source: F1000Res. 2024 Oct 31;10:1284. Originally published 2021 Dec 15. [Version 3] doi: 10.12688/f1000research.75116.3 (PMC11617828; doi:10.12688/f1000research.75116.3)
Supplement: Peer review history

# Reviewer report: Reviewer response for version 3

**DOI:** 10.5256/f1000research.167655.r336632

**Published:** 2024-12-04

**Authors:** Johannes van der Merwe

**Affiliations:** 1 KU Leuven, University Hospitals Leuven, Leuven, Belgium

No further comments. The authors responded to all my comments/queries and amended the manuscript where necessary.

Is the work clearly and accurately presented and does it cite the current literature?

Partly

If applicable, is the statistical analysis and its interpretation appropriate?

Yes

Are all the source data underlying the results available to ensure full reproducibility?

Partly

Is the study design appropriate and is the work technically sound?

Partly

Are the conclusions drawn adequately supported by the results?

Partly

Are sufficient details of methods and analysis provided to allow replication by others?

Yes

Reviewer Expertise:

Fetal medicine, placental development, preterm birth

I confirm that I have read this submission and believe that I have an appropriate level of expertise to confirm that it is of an acceptable scientific standard.

# Reviewer report: Reviewer response for version 2

**DOI:** 10.5256/f1000research.163402.r278020

**Published:** 2024-06-08

**Authors:** Johannes van der Merwe

**Affiliations:** 1 KU Leuven, University Hospitals Leuven, Leuven, Belgium

Comments for the Authors:

 This preclinical study aimed to evaluate the effect ethanol consumption during pregnancy in a mice model with/without IGF-1 deficiency. Overall, the topic is very relevant, the manuscript is well structured. However, there are some minor results reservations and multiple conclusions in the discussion is not supported by the results.

 Specific comments:

 1) Abstract

 Please define AAH in background section and then use abbreviation.

 --------------------------------------------------------------------------------------------------------

 2) Results:

 Section 3.1. Is it possible to give the IGF-1 serum levels data in table format. As it is unclear whether there are any differences between the relative control and ethanol groups.

 Section 3.2. Please give the data of the junctional zone comparisons.

 Section 3.3 Please give the absolute weight of the fetuses.

 --------------------------------------------------------------------------------------------------------

 3) Discussion:

 Paragraph 2 “Results in this project showed that ethanol consumption during gestation significantly affects IGF-1 serum levels in HZ pregnant dams, according to existing literature.” This statement is not supported by the results in 3.1 as there is no direct comparison between the HZ-controls and HZ-Ethanol. Please clarify.

 Paragraph 3. “The placenta is composed of by several strata, such as *myometrium*,…” The *myometrium* is not part of the placenta. Please rewrite.

 Paragraph 7. “Our results showed a decrease in AAH protein expression due to IGF-1 deficiency, mainly in the junctional zone…” and “Ethanol consumption throughout gestation promoted the arrest of trophoblasts in islets and also reduced AAH expression in the junctional zone of HZ and KO placentas” This statement is not supported by results in 3.4 as no quantification of the AAH expression is given in this section. Only representative images are given in Figure 4. Can the authors quantify the expression of AAH in order to make direct statistical comparisons?

 --------------------------------------------------------------------------------------------------------

 4) Conclusions:

 “…ethanol consumption during pregnancy promote placental disorganization, particularly in the junctional zone where trophoblast cells are expressed, and decrease placental efficiency, thus altering placental development and fetal growth.”

 The above statement does not seem to be supported by the results as shown. Since there is no quantification of junctional zone data and it was stated that there was no difference. Moreover, placental efficiently was only decreased in the KO group. It seems that this conclusion is overstated.

 The authors chose to quote the link to AAH protein expression but there is no quantifiable data given to support this statement. Furthermore, there was an increase in the mRNA expression for this protein. Please clarify or rewrite.

Is the work clearly and accurately presented and does it cite the current literature?

Partly

If applicable, is the statistical analysis and its interpretation appropriate?

Yes

Are all the source data underlying the results available to ensure full reproducibility?

Partly

Is the study design appropriate and is the work technically sound?

Partly

Are the conclusions drawn adequately supported by the results?

Partly

Are sufficient details of methods and analysis provided to allow replication by others?

Yes

Reviewer Expertise:

Fetal medicine, placental development, preterm birth

I confirm that I have read this submission and believe that I have an appropriate level of expertise to confirm that it is of an acceptable scientific standard, however I have significant reservations, as outlined above.

# Response

**Published:** 2024-06-17

**Authors:** Fabiola Castorena Torres

**Affiliations:** Tecnologico de Monterrey, Mexico

Monterrey, June 17 ^th^ 2024

 Dear Editorial Board members,

 First of all, I would like to thank you for your kind answer as for carefully revising the manuscript, that has greatly aimed us to improve our manuscript. Herein we will outline any changes made to the manuscript following reviewers’ advice (changes are shown in green in the manuscript) or, to the contrary, giving a rationale when a suggestion was not implemented.

**Reviewer 4:**

**This preclinical study aimed to evaluate the effect ethanol consumption during pregnancy in a mice model with/without IGF-1 deficiency. Overall, the topic is very relevant, the manuscript is well structured. However, there are some minor results reservations and multiple conclusions in the discussion is not supported by the results.**

 We would like to thank the reviewer for his/her comments, which have helped us to improve the manuscript.

**Specific comments** **:**

1. **Abstract:**

- **Please define AAH in background section and then use abbreviation.**

The reviewer is right. Sorry for this mistake, we have defined the abbreviation AAH.

1. **Results:**

- **Section 3.1. Is it possible to give the IGF-1 serum levels data in table format. As it is unclear whether there are any differences between the relative control and ethanol groups.**

The reviewer is right. We have included IGF-1 serum levels data in the Table.

- **Section 3.2. Please give the data of the junctional zone comparisons.**

As mentioned in section 3.2, we have included one figure as extended data (Figure 1) which shows all the placental zones analyzed in the present study: decidua, junctional zone and labyrinth. This figure is included in the following repository Zenodo: Ethanol consumption during gestation promotes placental alterations in IGF-1 deficient mice. https://doi.org/10.5281/zenodo.5750155

- **Section 3.3. Please give the absolute weight of the fetuses.**

The information about fetal weight is presented in the following article of our research group: “Martín-Estal, I., et al. (2022). Effect of Ethanol Consumption on the Placenta and Liver of Partially IGF-1- Deficient Mice: The Role of Metabolism via CYP2E1 and the Antioxidant Enzyme System. Biology, 11(9), 1264. https://doi.org/10.3390/biology11091264”.

1. **Discussion:**

- **Paragraph 2. “Results in this project showed that ethanol consumption during gestation significantly affects IGF-1 serum levels in HZ pregnant dams, according to existing literature.” This statement is not supported by the results in 3.1 as there is no direct comparison between the HZ-controls and HZ-Ethanol. Please clarify.**

In the present manuscript, serum from pregnant dams from the four experimental groups (WT-Control, WT-Ethanol, HZ-Control and HZ-Ethanol) was used to determine IGF-1 serum levels. As shown in Table 2 mentioned above, as expected, HZ-Control pregnant dams have lower IGF-1 serum levels compared to WT-Control dams. Additionally, ethanol consumption decreased even more IGF-1 serum levels than WT-Control. To obtain these results, all groups were compared (Mann-Whitney U test was used to compare between two groups, and Kruskall-Wallis test was used to compare between all experimental groups).

- **Paragraph 3. “The placenta is composed of by several strata, such as myometrium,…” The myometrium is not part of the placenta. Please rewrite.**

The reviewer is right. We have rewritten that sentence.

- **Paragraph 7. “Our results showed a decrease in AAH protein expression due to IGF-1 deficiency, mainly in the junctional zone…” and “Ethanol consumption throughout gestation promoted the arrest of trophoblasts in islets and also reduced AAH expression in the junctional zone of HZ and KO placentas” This statement is not supported by results in 3.4 as no quantification of the AAH expression is given in this section. Only representative images are given in Figure 4. Can the authors quantify the expression of AAH in order to make direct statistical comparisons?**

Thanks to the reviewer for this suggestion. We tried to measure quantitatively AAH expression in immunohistochemistry images, but unfortunately results were not conclusive. The results herein reported are only qualitative data. We have corrected these sentences as follows: “Our results suggested a decrease in AAH protein expression due to IGF-1 deficiency, mainly in the junctional zone, where trophoblast cells are located. Ethanol consumption throughout gestation seemed to promote the arrest of trophoblasts in islets and also reduced AAH expression in the junctional zone of HZ and KO placentas”.

1. **Conclusions:**

- **“…ethanol consumption during pregnancy promote placental disorganization, particularly in the junctional zone where trophoblast cells are expressed, and decrease placental efficiency, thus altering placental development and fetal growth.” The above statement does not seem to be supported by the results as shown. Since there is no quantification of junctional zone data and it was stated that there was no difference. Moreover, placental efficiently was only decreased in the KO group. It seems that this conclusion is overstated.**

The reviewer is right. We have corrected the sentence as follows: “ethanol consumption during pregnancy promote placental disorganization, particularly in the junctional zone where trophoblast cells are expressed, and decrease placental efficiency, especially in KO fetuses, thus altering placental development and fetal growth.”

- **The authors chose to quote the link to AAH protein expression but there is no quantifiable data given to support this statement. Furthermore, there was an increase in the mRNA expression for this protein. Please clarify or rewrite.**

The reviewer is right. Thanks for this suggestion. In spite of IGF-1 deficiency showed a significant increase in *Asph* expression in KO placentas from control group, while ethanol consumption during gestation also exhibited an increase in *Asph* in WT and HZ placentas; results for AAH protein expression in immunohistochemistry analyses suggested that both insults (IGF-1 deficiency and ethanol consumption) decreased AAH protein expression. However, immunohistochemistry analyses could not be quantified.

 This difference between mRNA and protein expression seems to point to the existence of a mechanism is taking place in order to promote IGF-1 downstream signaling expression to try to stimulate placental development despite IGF-1 deficiency and ethanol consumption. Nevertheless, more studies are needed in order to study this hypothesis, as suggested in the limitations section of the present manuscript.

 Yours faithfully,

 Inma Castilla de Cortázar Larrea, MD, PhD.

 Professor of Medical Physiology

 HM Velazquez Cabinet

 Velazquez 25, 28001 Madrid, Spain

 Tel.: +34 686652710

 Email: iccortazar@gmail.com

 Fabiola Castorena Torres, PhD.

 Research Professor, Coordinator of the Postgraduate Program in Biomedical Sciences

 Research and Innovation Department

 National School of Medicine and Health Sciences

 Tecnológico de Monterrey, México.

 Email: fcastorena@tec.mx

# Reviewer report: Reviewer response for version 2

**DOI:** 10.5256/f1000research.163402.r278024

**Published:** 2024-06-07

**Authors:** Lisa Akison, Sarah Steane

**Affiliations:** 1 The University of Queensland, South Brisbane, Australia

***General comments:***

 This study used a mouse model of maternal/paternal IGF1 partial deficiency (+/-) to investigate the effects of prenatal alcohol exposure on placental morphology and gene expression. This resulted in the full complement of genotypes (HZ +/-, KO -/- and WT +/+) for IGF1 in the fetal/placental pairs from each dam. The rationale for using this deficiency model was that alterations in IGF are proposed to be the underlying mechanism for alcohol-induced alterations in the placenta and subsequent growth restriction in the fetus. However, it is unclear how using a transgenic model with perturbed IGF1 expression in combination with alcohol exposure is more useful at determining mechanism than just measuring IGF1 protein/gene expression levels in an alcohol exposure model in WT mice. Indeed, the latter has already been done. While this study reports some potentially interesting observations regarding effects on placental morphology and gene expression, this is not supported by quantitative data. We also note that many of the comments raised by a previous reviewer have not been adequately addressed in the second version of the manuscript.

***Specific comments:***

- The previous reviewer requested that data for placental and fetal weights, as well as the fetal/placental ratio, be presented. The authors mention that this data is previously published (DOI: 10.3390/biology11091264 ) and yet there is no mention anywhere in the manuscript of this publication (although it has been tacked on at the end of the reference list with no citation in the text). This needs to be mentioned in the introduction to provide context for the current study.
- This previous study should also be mentioned in the methods when describing the animal treatments to provide important maternal/pregnancy information such as food and liquid intake, weight gain over gestation, average number of pups, sex ratio, as well as the fetal weight and placental weight mentioned above. This could simply be a description with a reference to the study with the original data.
- While the number of HZ dams that were ethanol vs control is provided in the methods (n=7 per group) it is unclear how many placentas were selected per dam for subsequent experiments. We reiterate the concerns made by the previous reviewer that the sample size for the WT control group is surprisingly small based on the expected ratio of HZ/WT/KO offspring. Also, the sample size indicated in the legend of Figure 3 for the KO control group was 22 and yet the supplemental file with raw data (and the subsequent study) only had a sample size of 19. The sample size calculation is also not optimal and a power analysis should have been used, particularly given that there has been a previous similar study done by the authors to estimate SD for this calculation.
- One of the major concerns of this study is that placental sex is not mentioned anywhere in the paper and is not factored into the analysis. There is a large body of evidence from both preclinical and clinical studies that the sex of the placenta influences adaptations to protect the fetus from maternal exposures, such as alcohol consumption. The responses to the previous reviewer indicate that fetal/placental sex was determined by the researchers. Indeed, the authors reveal that they only used male placentas in their analysis of gene expression to “reduce variability between groups”. They also reveal that a greater number of females were used for the HZ ethanol group in the data for figure 3. None of this is justified or mentioned in the methods for the paper.
- Another major concern is that representative images for immunohistochemistry of AAH and morphology of placentas are provided but there is no quantification to justify the claims in the text. Immunohistochemistry is best used to demonstrate localisation of protein in the tissue while Western Blot is required for quantification. The authors provide no evidence for the claims of decreases in AAH in the ethanol-exposed tissues. In fact, in response to a similar question by a previous reviewer, they mention that they did do quantification of their immunohistochemical staining but there were no significant differences, so data is not shown.
- Similarly, with respect to Figure 2, the authors claim in the text there is “…altered trophoblast migration and promoted placental hypoplasia, particularly in HZ and KO placentas”. However, there is no quantitative data to support this. The description of the tissues and cell types are also incorrect, as pointed out by the previous reviewer.
- The authors also claim that there were differences in gene expression due to ethanol treatment, but this is not justified by the data presented.
- The statistical analysis in Figure 3 is incorrect. The figure legend mentions that individual t-tests were used, but this should be analysed using a 2-way ANOVA with an appropriate post-hoc test (although the authors also mention that a GLIM was used but it is unclear if the P values come from this or the t-tests). There is also duplication of data from Figure 4G in Table 1, making the figure redundant.
- An interesting point was raised by the previous reviewer the different genotype for IGF-1 in decidua (maternal tissue) versus the fetus (i.e. that there is always partial deficiency in the decidua). The authors respond by suggesting a potential compensatory role of IGF2 but none of these points are discussed in the paper.

Is the work clearly and accurately presented and does it cite the current literature?

No

If applicable, is the statistical analysis and its interpretation appropriate?

No

Are all the source data underlying the results available to ensure full reproducibility?

Partly

Is the study design appropriate and is the work technically sound?

No

Are the conclusions drawn adequately supported by the results?

No

Are sufficient details of methods and analysis provided to allow replication by others?

No

Reviewer Expertise:

Animal models of prenatal alcohol exposure examining physiological, molecular and morphological outcomes in the placenta and offspring.

We confirm that we have read this submission and believe that we have an appropriate level of expertise to state that we do not consider it to be of an acceptable scientific standard, for reasons outlined above.

# Response

**Published:** 2024-06-17

**Authors:** Fabiola Castorena Torres

**Affiliations:** Tecnologico de Monterrey, Mexico

Monterrey, June 17 ^th^ 2024

 Dear Editorial Board members,

 First of all, I would like to thank you for your kind answer as for carefully revising the manuscript, that has greatly aimed us to improve our manuscript. Herein we will outline any changes made to the manuscript following reviewers’ advice (changes are shown in green in the manuscript) or, to the contrary, giving a rationale when a suggestion was not implemented.

**Reviewer 3:**

**This study used a mouse model of maternal/paternal IGF1 partial deficiency (+/-) to investigate the effects of prenatal alcohol exposure on placental morphology and gene expression. This resulted in the full complement of genotypes (HZ +/-, KO -/- and WT +/+) for IGF1 in the fetal/placental pairs from each dam. The rationale for using this deficiency model was that alterations in IGF are proposed to be the underlying mechanism for alcohol-induced alterations in the placenta and subsequent growth restriction in the fetus. However, it is unclear how using a transgenic model with perturbed IGF1 expression in combination with alcohol exposure is more useful at determining mechanism than just measuring IGF1 protein/gene expression levels in an alcohol exposure model in WT mice. Indeed, the latter has already been done. While this study reports some potentially interesting observations regarding effects on placental morphology and gene expression, this is not supported by quantitative data. We also note that many of the comments raised by a previous reviewer have not been adequately addressed in the second version of the manuscript.**

 We would like to thank the reviewer for his/her comments, which have helped us to improve the manuscript. Herein we will outline any changes made to the manuscript following reviewers’ advice or, to the contrary, giving a rationale when a suggestion was not implemented.

**Specific comments:**

- **The previous reviewer requested that data for placental and fetal weights, as well as the fetal/placental ratio, be presented. The authors mention that this data is previously published (DOI: 10.3390/biology11091264) and yet there is no mention anywhere in the manuscript of this publication (although it has been tacked on at the end of the reference list with no citation in the text). This needs to be mentioned in the introduction to provide context for the current study.**

The reviewer is right. It should be noted that the present manuscript belongs to a series of results of a larger project and it was first published in F1000 Research in December 2021. The second part of the results obtained from this project was published in August 2022 (DOI: 10.3390/biology11091264), the reason why there was no information about the data requested by the reviewers in the present manuscript. In this sense, we believe it would be inappropriate to cite an article that was published later than the present manuscript.

  

- **This previous study should also be mentioned in the methods when describing the animal treatments to provide important maternal/pregnancy information such as food and liquid intake, weight gain over gestation, average number of pups, sex ratio, as well as the fetal weight and placental weight mentioned above. This could simply be a description with a reference to the study with the original data.**

As mentioned before, it should be noted that the present manuscript belongs to a series of results of a larger project and it was first published in F1000 Research in December 2021. The second part of the results obtained from this project was published in August 2022 (DOI: 10.3390/biology11091264), the reason why there was no information about the data requested by the reviewers in the present manuscript. In this sense, we believe it would be inappropriate to cite an article that was published later than the present manuscript.

  

- **While the number of HZ dams that were ethanol vs control is provided in the methods (n=7 per group) it is unclear how many placentas were selected per dam for subsequent experiments. We reiterate the concerns made by the previous reviewer that the sample size for the WT control group is surprisingly small based on the expected ratio of HZ/WT/KO offspring. Also, the sample size indicated in the legend of Figure 3 for the KO control group was 22 and yet the supplemental file with raw data (and the subsequent study) only had a sample size of 19. The sample size calculation is also not optimal and a power analysis should have been used, particularly given that there has been a previous similar study done by the authors to estimate SD for this calculation.**

In the present study, the following four experimental groups were analyzed:

- WT-Control, *n = 6* pregnant dams.
- WT-Ethanol, *n = 4* pregnant dams.
- HZ-Control, *n = 6* pregnant dams.
- HZ-Ethanol, *n = 9* pregnant dams.

In the HZ-Ethanol group, a greater number of pregnant dams was used to decrease the risk of death or early delivery due to ethanol treatment. As mentioned by the reviewer, a low number of WT individuals were obtained in the HZ-Control group of pregnant dams, but nevertheless, Mendelian genetics is fulfilled.

 In the supplemental file with raw data only 4 placentas from each group were used to analyze gene expression by RT-PCR analysis.

- **One of the major concerns of this study is that placental sex is not mentioned anywhere in the paper and is not factored into the analysis. There is a large body of evidence from both preclinical and clinical studies that the sex of the placenta influences adaptations to protect the fetus from maternal exposures, such as alcohol consumption. The responses to the previous reviewer indicate that fetal/placental sex was determined by the researchers. Indeed, the authors reveal that they only used male placentas in their analysis of gene expression to “reduce variability between groups”. They also reveal that a greater number of females were used for the HZ ethanol group in the data for figure 3. None of this is justified or mentioned in the methods for the paper.**

Fetal sex was determined in the present study. However, only males were used to determine placental morphological alterations and gene expressions. As aforementioned, in the HZ-Ethanol group, a greater number of pregnant dams was used to decrease the risk of death or early delivery due to ethanol treatment.

- **Another major concern is that representative images for immunohistochemistry of AAH and morphology of placentas are provided but there is no quantification to justify the claims in the text. Immunohistochemistry is best used to demonstrate localisation of protein in the tissue while Western Blot is required for quantification. The authors provide no evidence for the claims of decreases in AAH in the ethanol-exposed tissues. In fact, in response to a similar question by a previous reviewer, they mention that they did do quantification of their immunohistochemical staining but there were no significant differences, so data is not shown.**

Thanks to the reviewer for this suggestion. First of all, we tried to measure AAH protein expression in placenta by Western Blot analysis, but no results were obtained, because the amount of sample was not sufficient to obtain results. Also, we tried to measure quantitatively AAH expression in immunohistochemistry images, but unfortunately results were not conclusively. The results herein reported are only qualitative data. We have corrected the discussion section as follows: “Our results suggested a decrease in AAH protein expression due to IGF-1 deficiency, mainly in the junctional zone, where trophoblast cells are located. Ethanol consumption throughout gestation seemed to promote the arrest of trophoblasts in islets and also reduced AAH expression in the junctional zone of HZ and KO placentas”.

- **Similarly, with respect to Figure 2, the authors claim in the text there is “…altered trophoblast migration and promoted placental hypoplasia, particularly in HZ and KO placentas”. However, there is no quantitative data to support this. The description of the tissues and cell types are also incorrect, as pointed out by the previous reviewer.**

The reviewer is right. Although there are findings that could indicate the presence of placental hypoplasia (e.g., placental weight), more studies are needed to reach this conclusion. We removed this sentence.

- **The authors also claim that there were differences in gene expression due to ethanol treatment, but this is not justified by the data presented.**

The reviewer is right. In the present study, when statistical analysis was performed, first we grouped the results by treatment (Water -control- or ethanol), and significant results were obtained ( *p < 0.05*). However, when the results were grouped by genotype (WT, HZ and KO), no significant differences were observed.

- **The statistical analysis in Figure 3 is incorrect. The figure legend mentions that individual t-tests were used, but this should be analysed using a 2-way ANOVA with an appropriate post-hoc test (although the authors also mention that a GLIM was used but it is unclear if the P values come from this or the t-tests). There is also duplication of data from Figure 4G in Table 1, making the figure redundant.**

Thanks to the reviewer for his/her suggestion. ANOVA analyzes the magnitude of the effect, while general linear model analyzes both the magnitude and the direction of the effect. In this case, to better evaluate the effect of the two independent variables in the present study (ethanol consumption and IGF-1 deficiency) a general linear model was performed. In this sense, the p-values obtained belong to both statistical analyses, considering significant differences at a level of *p < 0.05*.

 In Table 1, we present the results from RT-PCR analyses, having Asph expression statistical significance. For this reason, these results were the only ones that were represented graphically, in order to better visualize the results.

- **An interesting point was raised by the previous reviewer the different genotype for IGF-1 in decidua (maternal tissue) versus the fetus (i.e. that there is always partial deficiency in the decidua). The authors respond by suggesting a potential compensatory role of IGF2 but none of these points are discussed in the paper.**

The reviewer is right, thanks for his/her suggestion. The present manuscript focuses mainly on IGF-1. However, we have included the following sentence: “Although the decidua, which is in contact with the maternal side, is always partially deficient in IGF-1, the rest of the placenta depends on the fetus; that is, if the fetus is WT, the placenta will be of the same genotype. Also, IGF-2 has an important role in fetal growth due to its direct anabolic actions within the fetus, and by its modulation of placental supply capacity, which may be a compensatory mechanism for IGF-1 deficiency (10.1016/j.placenta.2018.01.005).”

 Yours faithfully,

 Inma Castilla de Cortázar Larrea, MD, PhD.

 Professor of Medical Physiology

 HM Velazquez Cabinet

 Velazquez 25, 28001 Madrid, Spain

 Tel.: +34 686652710

 Email: iccortazar@gmail.com

 Fabiola Castorena Torres, PhD.

 Research Professor, Coordinator of the Postgraduate Program in Biomedical Sciences

 Research and Innovation Department

 National School of Medicine and Health Sciences

 Tecnológico de Monterrey, México.

 Email: fcastorena@tec.mx

# Reviewer report: Reviewer response for version 2

**DOI:** 10.5256/f1000research.163402.r278027

**Published:** 2024-05-22

**Authors:** Berthold Huppertz

**Affiliations:** 1 Division of Cell Biology, Histology & Embryology; Gottfried Schatz Research Center, Medical University of Graz, Graz, Austria

The article of Martín-Estal et al. presents data on the effect of ethanol consumption during pregnancy in the mouse. In parallel, the authors knocked-out IGF-1 in the dams to generate WT, HZ and KO fetuses and placentas. This is an interesting study looking into the double effect of IGF-1 deficiency and ethanol consumption. This reviewer has identified quite a number of specific concerns, see below for details.

 General concerns

 Please add the species to the respective presentation of published data throughout the manuscript, e.g. page 4, 2 ^nd^ paragraph.

 Specific concerns

 Title: Please provide the species in the title. This always helps the reader to directly assign the study to the correct field. E.g.: “… in IGF-1 deficient *MOUSE PLACENTAS*”

 Abstract, background: Please define the abbreviation AAH here.

 Abstract, methods: Please better define what is meant with *“… were given 10% ethanol …*”. Was this 10% ethanol in water or in food or both? Please specify.

 P4, 4 ^th^ paragraph: Here the authors state that “ *AAH is overexpressed in trophoblast cells*”. For this reviewer the word “overexpressed” points to a non-physiologically high expression of the protein. Since this is not the case, this reviewer would recommend to use a different wording, such as “AAH is HIGHLY expressed in trophoblast cells”.

 P4, 9 ^th^ paragraph: The 10% ethanol were only given in water? If so, was water changed every day? Did the authors took the evaporation of ethanol over time into account? Have the authors monitored how much water was used? If so, were there differences between water and 10% ethanol?

 P5, 2 ^nd^ paragraph: Here the authors state: “ *a caesarean section was performed to obtain fetuses and placentas, which were measured and weighted (Figure 1)*.” However, fetuses, measurements and weights cannot be found in figure 1. Figure 3 shows ratios, while the original values are missing.

 P5, 2rd paragraph: What is meant with “ *Fetuses and placentas were stored randomly blind*”? Please explain. The authors should know to which group the fetuses/placentas belong and what the fetus-placenta pairs are.

 P5, 2.3: Only here the authors tell the reader that WT mice were treated with ethanol as well. Please extend chapter 2.1 and clearly describe the groups and treatments. Also, it does not become clear whether the WT mice were pregnant as well. Please specify.

 P5, 2.3: Please clearly describe which sera were used for IGF-1 Elisas. In the moment, the description is unintelligible (first sentence of 2.3).

 P6, 1 ^st^ paragraph: Here the authors describe that “ *All images were analysed using the processing package ImageJ* …”. Please describe the way images were taken (Randomly? Systematic randomly? Magnification? Number of images per placenta? etc.)

 P6, 3 ^rd^ paragraph: Here the authors describe that “ *Digital images of tissue sections (five images per placenta) were captured by three observers (double-blind)* …”. What does this mean? Was there a total of 15 images per slide or only 5? How did the observers choose the images? What was the magnification of the images?

 P6, 3.1: This reviewer does not understand the last sentence of this chapter: “ *Interestingly, a possible synergic effect, that decreased IGF-1 serum levels, was observed by the partial IGF-1 deficiency and ethanol consumption (HZ-Ethanol) during gestation compared to WT-Control group (p < 0.01).*” The IGF-1 values for the HZ groups are not different and the IGF-1 values for the WT groups are also not different (each w and w/o ethanol). And of course, the IGF-1 values in the HZ groups are significantly reduced compared to the WT groups. Where do the authors see a synergistic effect of IGF-1 deficiency and ethanol? What about the serum IGF-1 values in the KO groups?

 P7, 3.2: In the first sentence, the authors refer to figure 1 for extended data on the strata of the placenta. However, this information is not given in figure 1.

 P7, 3.2: Only now the authors provide the information that there were pregnant WT animals. Please add their numbers, age, placental and fetal weights, number of pups etc. to the methods section. Best would be to provide a table with the HZ and WT animals and their numbers, weights (fetal and placental), etc.

 P7, 3.2 & figure 2: The text reads as if figure 2A is derived from a WT placenta of a WT mouse (“ *In the WT placentas from the control group …*”). If this is the case, this needs to be clarified in the caption of figure 2. Then, figure 2 needs an additional row of images with the WT placentas from HZ dams.

 P7, 3.2: If the wording above (“ *In the WT placentas from the control group …*”) points to WT dams with WT fetuses, then he question arises what the following animals are: “ *KO placentas from the control group*”. This is hard to understand. Please explain.

 P7-8, 3.2: Here the authors state that ethanol consumption “ *promoted trophoblast arrest in islets in all groups*”. How do the authors know there is arrest of trophoblast cells? The “islets” may well be finger-like structures of the junctional zone reaching into the labyrinth, pointing to a less organized layering of labyrinth and junctional zone.

 P8, 3.2: Here the authors state that “ *ethanol use altered trophoblast migration (Figure 2D) and promoted placental hypoplasia*”. Questions: (1) How did the authors assess differences in trophoblast migration from these images? (2) Where did the authors identify placental hypoplasia? From the images in figure 2, it looks as if the area of the labyrinth is increased with ethanol, at least in WT and HZ.

 P8, 3.3: Here the authors state that “The chronic exposure to ethanol during gestation resulted in a significant reduction in placental efficiency (p < 0.001) compared to control groups.”. Please add any value to support this statement. As can be seen from figure 3, only in the KO group there was a significant (<0.05) reduction in the fetal/placental weight ratio.

 P8, 3.3: Idea: It looks as if the placenta tried to counteract the reduced fetal growth under ethanol. The increased area of the labyrinth points to this. Hence, could the authors check volumes/areas of the labyrinth between groups?

 P8, ,3.4: Here the authors state that “ *IGF-1 deficiency revealed a decrease in AAH expression within the junctional zone …*”. Such a statement should only be made if there is a respective analysis with a p<0.05. However, there are only a few images that have not been quantitatively analyzed.

 P9, 3.4, last sentence (“ *Ethanol consumption …*”): The statement that ethanol decreased AAH expression is not supported by respective data. Protein has not been quantified and RNA does not show differences. The rest of the sentence is mere speculation without supporting data, so please remove or rephrase.

 P10, 3 ^rd^ paragraph of discussion: The junctional zone of the mouse placenta is NOT composed of syncytiotrophoblast and cytotrophoblast, this is only true for the labyrinth. Please correct.

 P10, 3 ^rd^ paragraph of discussion: Since the authors have wrongly allocated the syncytiotrophoblast (as major source for IGF-1) to the junctional zone, their concept of reduced expression of IGF-1 in the junctional zone has lost its scientific basis. See also p12, lines 5-8.

 P10, 4 ^th^ paragraph of discussion: The authors do not show data to support placental hypoplasia or changes in trophoblast motility and trophoblast invasion. Hence, the respective discussion should be written with great caution.

 Figure 1: This is a nice representation of the experimental procedure. This could be further improved by adding the following: (1) Add the number of pregnant animals per group. (2) Since the HZ animals were given ethanol during pregnancy 8 or not), this could be indicated as well. (3) The WT animals need to be added as well. (4) The number of placentas in the three groups can be added.

 Figure 2: The comparison of images in A and D (WT w and w/o ethanol) shows a massively increased area of the labyrinth. Is this only present in this one case or has this been a general feature of ethanol in the WT mice? A similar difference can be found in the HZ/HZ mice (2B/E).

 Figure 4: Just a comment: The data from the analysis of the AAH protein (A-F) do not fit the data from the analysis of the AAH RNA (G). This is not surprising as differences between RNA and protein expression are regularly found.

Is the work clearly and accurately presented and does it cite the current literature?

Yes

If applicable, is the statistical analysis and its interpretation appropriate?

Yes

Are all the source data underlying the results available to ensure full reproducibility?

Yes

Is the study design appropriate and is the work technically sound?

Partly

Are the conclusions drawn adequately supported by the results?

No

Are sufficient details of methods and analysis provided to allow replication by others?

Partly

Reviewer Expertise:

Placenta; trophoblast; cell biology

I confirm that I have read this submission and believe that I have an appropriate level of expertise to confirm that it is of an acceptable scientific standard, however I have significant reservations, as outlined above.

# Response

**Published:** 2024-06-06

**Authors:** Fabiola Castorena Torres

**Affiliations:** Tecnologico de Monterrey, Mexico

Monterrey, June 5 ^th^ 2024

 Dear Editorial Board members,

 First of all, I would like to thank you for your kind answer as for carefully revising the manuscript, that has greatly aimed us to improve our manuscript. Herein we will outline any changes made to the manuscript following reviewers’ advice (changes are shown in green in the manuscript) or, to the contrary, giving a rationale when a suggestion was not implemented.

**Reviewer 2:**

**The article of Martín-Estal et al. presents data on the effect of ethanol consumption during pregnancy in the mouse. In parallel, the authors knocked-out IGF-1 in the dams to generate WT, HZ and KO fetuses and placentas. This is an interesting study looking into the double effect of IGF-1 deficiency and ethanol consumption. This reviewer has identified quite a number of specific concerns, see below for details.**

 We would like to thank the reviewer for his/her comments, which have helped us to improve the manuscript.

**General concerns:**

**Please add the species to the respective presentation of published data throughout the manuscript, e.g. page 4, 2nd paragraph.**

 The reviewer is right. We added this information.

**Specific concerns:**

- **Title** **: Please provide the species in the title. This always helps the reader to directly assign the study to the correct field. E.g.: “… in IGF-1 deficient MOUSE PLACENTAS”.**

The reviewer is right. We have changed the title as follows: “Ethanol consumption during gestation promotes placental alterations in IGF-1 deficient mouse placentas”.

- **Abstract, background** **: Please define the abbreviation AAH here.**

The reviewer is right. Sorry for this mistake, we have defined the abbreviation AAH.

- **Abstract, methods** **: Please better define what is meant with “… were given 10% ethanol …”. Was this 10% ethanol in water or in food or both? Please specify.**

The reviewer is right. Mice were given 10% ethanol in water during the experimental protocol. We have added this information in the abstract section. In the methods sections, this information was specified: “An adaptation period to ethanol (14 days) was performed, where HZ female mice were given increasing concentrations of 2%, 5% and 10% of ethanol in water” (Kleiber ML, Wright E, Singh SM: Maternal voluntary drinking in C57BL/6J mice: Advancing a model for fetal alcohol spectrum disorders. Behav. Brain Res. 2011;223:376–387. Elsevier B.V. 21601595 10.1016/j.bbr.2011.05.005).

  

- **P4, 4th paragraph** **: Here the authors state that “AAH is overexpressed in trophoblast cells”. For this reviewer the word “overexpressed” points to a non-physiologically high expression of the protein. Since this is not the case, this reviewer would recommend to use a different wording, such as “AAH is HIGHLY expressed in trophoblast cells”.**

The reviewer is right. Sorry for this mistake, we have changed this word as suggested.

- **P4, 9th paragraph** **: The 10% ethanol were only given in water? If so, was water changed every day? Did the authors took the evaporation of ethanol over time into account? Have the authors monitored how much water was used? If so, were there differences between water and 10% ethanol?**

The 10% ethanol was only given in water, and this water was changed every day for all mice included in the ethanol group. We have considered the evaporation of ethanol over time, but no significant differences were observed between ethanol groups and control groups (water).

- **P5, 2nd paragraph** **: Here the authors state: “a caesarean section was performed to obtain fetuses and placentas, which were measured and weighted (Figure 1).” However, fetuses, measurements and weights cannot be found in figure 1. Figure 3 shows ratios, while the original values are missing.**

The reviewer is right. We removed “Figure 1” from this statement. This information about fetal parameters (fetal weight, fetal height and placental weight) is presented in the following article of our research group: “Martín-Estal, I., et al. (2022). Effect of Ethanol Consumption on the Placenta and Liver of Partially IGF-1- Deficient Mice: The Role of Metabolism via CYP2E1 and the Antioxidant Enzyme System. Biology, 11(9), 1264. https://doi.org/10.3390/biology11091264”.

- **P5, 2rd paragraph** **: What is meant with “Fetuses and placentas were stored randomly blind”? Please explain. The authors should know to which group the fetuses/placentas belong and what the fetus-placenta pairs are.**

After cesarean section, fetuses an placentas from pregnant dams from different groups were stored in paraformaldehyde 4% or liquid nitrogen. The determination of which set of fetuses and placentas from a pregnant dam from an experimental group was determined randomly for each assay, but we know which group the fetuses/placentas belong and what the fetus-placenta pairs are. We removed “blind” from this statement.

- **P5, 2.3** **: Only here the authors tell the reader that WT mice were treated with ethanol as well. Please extend chapter 2.1 and clearly describe the groups and treatments. Also, it does not become clear whether the WT mice were pregnant as well. Please specify.**

The reviewer is right. We are sorry for this mistake. In the present project, four experimental groups of pregnant dams were analyzed: two groups given water (WT-Control and HZ-Control), and two groups given 10% ethanol in water (WT-Ethanol and HZ-Ethanol). For subsequent analysis, only fetuses and placentas from HZ x HZ matings were analyzed. We have included this information in the manuscript.

- **P5, 2.3** **: Please clearly describe which sera were used for IGF-1 Elisas. In the moment, the description is unintelligible (first sentence of 2.3).**

The reviewer is right. We are sorry for this mistake. As mentioned above, in the present project, four experimental groups of pregnant dams were analyzed: two groups given water (WT-Control and HZ-Control), and two groups given 10% ethanol in water (WT-Ethanol and HZ-Ethanol). Serum from pregnant dams from these four experimental groups were used to determine IGF-1 values.

- **P6, 1st paragraph** **: Here the authors describe that “All images were analysed using the processing package ImageJ …”. Please describe the way images were taken (Randomly? Systematic randomly? Magnification? Number of images per placenta? etc.).**

In the placental histological study section, this information is included in the present manuscript: “Finally, all histological preparations were evaluated independently by three observers (double-blind) in a Zeiss Axio Imager M2 microscope (Zeiss, Baden-Württemberg, Germany) to establish morphology alterations in the placenta due to ethanol consumption throughout gestation.” Also, this statement is included: “Three images were taken per sample of at least three individuals from each group and genotype.”

 In Figure 2, this information is included in the figure legend: “Original magnification 100x. Scale bar = 100 μm.”

 In the immunohistochemical staining section, this information is included in the present manuscript: “Digital images of tissue sections (five images per placenta) were analyzed by three observers (double-blind) using a Zeiss Axio Imager M2 microscope. All images were analysed using the processing package ImageJ (National Institutes of Health, Maryland, USA).”

 In Figure 4, this information is included in the figure legend: “Original magnification 100x.” We have included the following information: “Scale bar = 100 μm.”

- **P6, 3rd paragraph** **: Here the authors describe that “Digital images of tissue sections (five images per placenta) were captured by three observers (double-blind) …”. What does this mean? Was there a total of 15 images per slide or only 5? How did the observers choose the images? What was the magnification of the images?**

In the immunohistochemical staining section, five images per placenta were captured and then were analyzed by three observers (double-blind). The reviewer is right, we changed the sentence.

 The magnification of the images is included in the legend of figure 4.

- **P6, 3.1** **: This reviewer does not understand the last sentence of this chapter: “Interestingly, a possible synergic effect, that decreased IGF-1 serum levels, was observed by the partial IGF-1 deficiency and ethanol consumption (HZ-Ethanol) during gestation compared to WT-Control group (p < 0.01).” The IGF-1 values for the HZ groups are not different and the IGF-1 values for the WT groups are also not different (each w and w/o ethanol). And of course, the IGF-1 values in the HZ groups are significantly reduced compared to the WT groups. Where do the authors see a synergistic effect of IGF-1 deficiency and ethanol? What about the serum IGF-1 values in the KO groups?**

To validate IGF-1 serum levels in the present experimental model, serum from pregnant dams from the four experimental groups were used (WT-Control, WT-Ethanol, HZ-Control and HZ-Ethanol). As shown in the following table, as expected, HZ-Control pregnant dams have lower IGF-1 serum levels compared to WT-Control dams. Ethanol consumption decreased even more IGF-1 serum levels than WT-Control.

**WT-Control: **739 ± 48 ng/mL IGF-1 serum levels

**WT-Ethanol: **795 ± 147 ng/mL IGF-1 serum levels

**HZ-Control: **454 ± 58 ^*, a^ ng/mL​​​​​​ ​IGF-1 serum levels

**HZ-Ethanol: **422 ± 43 ^*, b^ ng/mL​​​​​​ IGF-1 serum levels

^*^ *p < 0.001* HZ vs WT.

^a^ *p < 0.005* HZ-Control vs WT-Control.

^b^ *p < 0.01* HZ-Ethanol vs WT-Control.

- **P7, 3.2** **: In the first sentence, the authors refer to figure 1 for extended data on the strata of the placenta. However, this information is not given in figure 1.**

We have included one figure as extended data (Figure 1) which shows all the placental zone analyzed in the present study. This figure is included in the following repository Zenodo: Ethanol consumption during gestation promotes placental alterations in IGF-1 deficient mice. https://doi.org/10.5281/zenodo.5750155 52.

- **P7, 3.2** **: Only now the authors provide the information that there were pregnant WT animals. Please add their numbers, age, placental and fetal weights, number of pups etc. to the methods section. Best would be to provide a table with the HZ and WT animals and their numbers, weights (fetal and placental), etc.**

The reviewer is right. We are sorry for this mistake. In the present project, four experimental groups of pregnant dams were analyzed: two groups given water (WT-Control and HZ-Control), and two groups given 10% ethanol in water (WT-Ethanol and HZ-Ethanol). For subsequent analysis, only fetuses and placentas from HZ x HZ matings were analyzed. We have included this information in the manuscript nad in Figure 1. The other data suggested by the reviewer can be found in the following article from our research group: “Martín-Estal, I., et al. (2022). Effect of Ethanol Consumption on the Placenta and Liver of Partially IGF-1- Deficient Mice: The Role of Metabolism via CYP2E1 and the Antioxidant Enzyme System. Biology, 11(9), 1264. https://doi.org/10.3390/biology11091264”.

- **P7, 3.2 & figure 2** **: The text reads as if figure 2A is derived from a WT placenta of a WT mouse (“In the WT placentas from the control group …”). If this is the case, this needs to be clarified in the caption of figure 2. Then, figure 2 needs an additional row of images with the WT placentas from HZ dams.**

The images shown in figure 2 belong to placentas from HZ-Control and HZ-Ethanol groups. These groups were matted with HZ male mice (HZ x HZ), resulting in three different fetal genotypes: WT, HZ and KO. In this case, WT, HZ and KO placentas correspond to fetal placentas from HZ-Control and HZ-Ethanol groups.

- **P7, 3.2** **: If the wording above (“In the WT placentas from the control group …”) points to WT dams with WT fetuses, then he question arises what the following animals are: “KO placentas from the control group”. This is hard to understand. Please explain.**

The wording “In the WT placentas from the control group…” belongs to placentas from HZ-Control group. Sorry for these misunderstandings due to the nomenclature.

- **P7-8, 3.2** **: Here the authors state that ethanol consumption “promoted trophoblast arrest in islets in all groups”. How do the authors know there is arrest of trophoblast cells? The “islets” may well be finger-like structures of the junctional zone reaching into the labyrinth, pointing to a less organized layering of labyrinth and junctional zone.**

The reviewer is right. However, as it can be seen in Figure 2, trophoblast are arrested in island-like forms, denoting poor organization and development of the placenta.

- **P8, 3.2** **: Here the authors state that “ethanol use altered trophoblast migration (Figure 2D) and promoted placental hypoplasia”. Questions: (1) How did the authors assess differences in trophoblast migration from these images? (2) Where did the authors identify placental hypoplasia? From the images in figure 2, it looks as if the area of the labyrinth is increased with ethanol, at least in WT and HZ.**

The reviewer is right. Although there are findings that could indicate the presence of placental hypoplasia (e.g., placental weight), more studies are needed to reach this conclusion. We removed this sentence.

- **P8, 3.3** **: Here the authors state that “The chronic exposure to ethanol during gestation resulted in a significant reduction in placental efficiency (p < 0.001) compared to control groups.”. Please add any value to support this statement. As can be seen from figure 3, only in the KO group there was a significant (<0.05) reduction in the fetal/placental weight ratio.**

This statement corresponds to the comparison between both ethanol and control groups, proving that ethanol first reduces placental efficiency. Then, a multiple comparison (general linear model) between all genotypes from all experimental groups were performed, in order to validate if IGF-1 deficiency promotes this alteration in placental efficiency.

- **P8, 3.3** **: Idea: It looks as if the placenta tried to counteract the reduced fetal growth under ethanol. The increased area of the labyrinth points to this. Hence, could the authors check volumes/areas of the labyrinth between groups?**

The reviewer is right. This is an interesting suggestion, but we could not be able to measure the volumes/areas of the placentas. This measurement would be very useful to understand and explain the ethanol’s teratogenic effects during gestation.

- **P8, 3.4** **: Here the authors state that “IGF-1 deficiency revealed a decrease in AAH expression within the junctional zone …”. Such a statement should only be made if there is a respective analysis with a p<0.05. However, there are only a few images that have not been quantitatively analyzed.**

We tried to measure quantitatively AAH expression in immunohistochemistry images, but unfortunately results were not conclusively. The results herein reported are only qualitative data.

- **P9, 3.4, last sentence (“Ethanol consumption …”):** **The statement that ethanol decreased AAH expression is not supported by respective data. Protein has not been quantified and RNA does not show differences. The rest of the sentence is mere speculation without supporting data, so please remove or rephrase.**

The reviewer is right. We have removed this sentence.

- **P10, 3rd paragraph of discussion** **: The junctional zone of the mouse placenta is NOT composed of syncytiotrophoblast and cytotrophoblast, this is only true for the labyrinth. Please correct.**

Thanks for the reviewer’s comments. Trophoblast giant cells, the invasive cells responsible for invading maternal vasculature to promote placental development, are located in the labyrinth. However, spongiotrophoblast cells and glycogen trophoblast cells are found in the labyrinth and in the junctional zone (10.3390/nu15163564).

- **P10, 3rd paragraph of discussion** **: Since the authors have wrongly allocated the syncytiotrophoblast (as major source for IGF-1) to the junctional zone, their concept of reduced expression of IGF-1 in the junctional zone has lost its scientific basis. See also p12, lines 5-8.**

Thanks for the reviewer’s comments. As aforementioned, trophoblast giant cells, the invasive cells responsible for invading maternal vasculature to promote placental development, are located in the labyrinth. However, spongiotrophoblast cells and glycogen trophoblast cells are found in the labyrinth and in the junctional zone (10.3390/nu15163564). Throphoblast invasion is observed in the junctional zone, in order to develop a normal placenta. Alterations in this trophoblast migration can lead to adverse pregnancy consequences due to a limit blood flow, such as fetal growth restriction and preeclampsia (10.1067/mob.2002.127305).

- **P10, 4th paragraph of discussion** **: The authors do not show data to support placental hypoplasia or changes in trophoblast motility and trophoblast invasion. Hence, the respective discussion should be written with great caution.**

The reviewer is right. Although there are findings that could indicate the presence of placental hypoplasia (e.g., placental weight), more studies are needed to reach this conclusion. We removed this sentence.

- **Figure 1** **: This is a nice representation of the experimental procedure. This could be further improved by adding the following: (1) Add the number of pregnant animals per group. (2) Since the HZ animals were given ethanol during pregnancy 8 or not), this could be indicated as well. (3) The WT animals need to be added as well. (4) The number of placentas in the three groups can be added.**

The reviewer is right. We have updated Figure 1 as suggested.

- **Figure 2** **: The comparison of images in A and D (WT w and w/o ethanol) shows a massively increased area of the labyrinth. Is this only present in this one case or has this been a general feature of ethanol in the WT mice? A similar difference can be found in the HZ/HZ mice (2B/E).**

This increased area of the labyrinth was presented in most of the cases, suggesting that this increase in the area may be due to placental disorganization caused by ethanol consumption.

- **Figure 4** **: Just a comment: The data from the analysis of the AAH protein (A-F) do not fit the data from the analysis of the AAH RNA (G). This is not surprising as differences between RNA and protein expression are regularly found.**

The reviewer is right. One of the limitations of the study is that we could not measure AAH protein expression levels in placenta, results that can be useful to explain the ethanol teratogenic effects during gestation and their relation with IGF-1 deficiency.

 Yours faithfully,

 Inma Castilla de Cortázar Larrea, MD, PhD.

 Professor of Medical Physiology

 HM Velazquez Cabinet

 Velazquez 25, 28001 Madrid, Spain

 Tel.: +34 686652710

 Email: iccortazar@gmail.com

 Fabiola Castorena Torres, PhD.

 Research Professor, Coordinator of the Postgraduate Program in Biomedical Sciences

 Research and Innovation Department

 National School of Medicine and Health Sciences

 Tecnológico de Monterrey, México.

 Email: fcastorena@tec.mx

# Reviewer report: Reviewer response for version 1

**DOI:** 10.5256/f1000research.78941.r199303

**Published:** 2023-10-03

**Authors:** Ludivine Doridot

**Affiliations:** 1 Institut Cochin, Inserm, CNRS, F-75014, Cite Internationale Universitaire de Paris, Paris, Île-de-France, France

General comment:

 Martín-Estal et al. performed a well-planned study to evaluate the effect of ethanol consumption on placental development (and fetal growth) in mice partially deficient for IGF1, as well as a potential synergy between (partial or total) IGF-1 deficiency and ethanol consumption on placental development. They notably showed that total IGF-1 deficiency and ethanol consumption both led to structural anomalies in the placental layers. They also showed a reduced placental efficiency in IGF-1 KO placentas, that was further reduced by ethanol consumption. They also evaluated the mRNA and protein expression of AAH and the mRNA expression of a few other genes.

 While the data are interesting, their description and analysis is not sufficient. The fetal weight is an important element and thus should be presented. Fetal growth retardation/restriction is indeed a key aspect of IGF-1 signaling and ethanol consumption, as it is well described in the introduction. I would suggest adding 2 panels to Figure 3 with the fetal and placental weight (in addition to the ratio), this is especially important as the authors mentioned placental hypoplasia that is not visible in the presented data. The placental morphology should be analyzed more thoroughly with for example a quantification of the length ratio between JZ/labyrinth and/or the number of abnormal islets in the labyrinth (the quality of the image should also be improved as the text within the image is hardly readable). The AAH immunohistochemistry would also benefit for a quantification analysis (in specific layers). For all the quantitative data, the statistical analysis effectively used should be described with the indication of the number of analyzed samples per group. Post hoc test should be performed after two-way ANOVA or Kruskal Wallis test, to compare the genotypes within the water or ethanol group, and compare the effect of ethanol within each genotype. Only with these additional analyses will it be possible to conclude more clearly on the results.

 More specific comments:

 In Figure 3, there are Control: WT, n = 9; HZ, n = 32; KO, n = 22; Ethanol: WT, n = 23; HZ, n = 44; KO, n = 16. This does not fit with the expected ratio of 25/50/25% of WT/HZ/KO. It seems that there are some missing control WT. Could the authors explain this?

 And for the RT-qPCR, it does not seem that all these placentas were analyzed. How were the one included chosen?

 Syncytiotrophoblast are in the labyrinth layer and not the junctional zone (see for example 10.3389/fimmu.2021.771054). The myometrium is not part of the placenta.

 For clarity, I would suggest to use Igf+/+, Igf +/- and Igf-/- rather than WT, HZ and KO throughout the manuscript.

 The author should also discuss the fact that the decidua is always partially deficient in IGF1 which may influence placental establishment, as well as the disparity of mice age that could also influence the results.

 Did the ethanol consumption influence the weight gain of the mice throughout the study?

 How many HZ-ethanol female were actually mated?

 The title should be : Ethanol consumption during gestation promotes placental alterations in IGF-1 deficient placentas

 Is the fetal weight further reduced in Ethanol KO compared to water-KO?

Is the work clearly and accurately presented and does it cite the current literature?

Partly

If applicable, is the statistical analysis and its interpretation appropriate?

Partly

Are all the source data underlying the results available to ensure full reproducibility?

Yes

Is the study design appropriate and is the work technically sound?

Yes

Are the conclusions drawn adequately supported by the results?

Partly

Are sufficient details of methods and analysis provided to allow replication by others?

Partly

Reviewer Expertise:

Reproductive biology

I confirm that I have read this submission and believe that I have an appropriate level of expertise to confirm that it is of an acceptable scientific standard, however I have significant reservations, as outlined above.

# Response

**Published:** 2024-03-04

**Authors:** Fabiola Castorena Torres

**Affiliations:** Tecnologico de Monterrey, Mexico

Monterrey, March 4 ^th^ 2024

 Dear Editorial Board members,

 First of all, I would like to thank you for your kind answer as for carefully revising the manuscript, that has greatly aimed us to improve our manuscript. Herein we will outline any changes made to the manuscript following reviewers’ advice (changes are shown in green in the manuscript) or, to the contrary, giving a rationale when a suggestion was not implemented.

**Reviewer 1:**

**Martín-Estal et al. performed a well-planned study to evaluate the effect of ethanol consumption on placental development (and fetal growth) in mice partially deficient for IGF1, as well as a potential synergy between (partial or total) IGF-1 deficiency and ethanol consumption on placental development. They notably showed that total IGF-1 deficiency and ethanol consumption both led to structural anomalies in the placental layers. They also showed a reduced placental efficiency in IGF-1 KO placentas, that was further reduced by ethanol consumption. They also evaluated the mRNA and protein expression of AAH and the mRNA expression of a few other genes.**

 We would like to thank the reviewer for his/her comments, which have helped us to improve the manuscript.

**While the data are interesting, their description and analysis is not sufficient.**

- **The fetal weight is an important element and thus should be presented. Fetal growth retardation/restriction is indeed a key aspect of IGF-1 signaling and ethanol consumption, as it is well described in the introduction. **

The reviewer is right. This information about fetal parameters (fetal weight, fetal height and placental weight) is presented in the following article of our research group: “Martín-Estal, I., *et al.* (2022). Effect of Ethanol Consumption on the Placenta and Liver of Partially IGF-1-Deficient Mice: The Role of Metabolism via CYP2E1 and the Antioxidant Enzyme System. Biology, 11(9), 1264. https://doi.org/10.3390/biology11091264”.

- **I would suggest adding 2 panels to Figure 3 with the fetal and placental weight (in addition to the ratio), this is especially important as the authors mentioned placental hypoplasia that is not visible in the presented data. **

The reviewer is right. Information is presented in the following article of our research group: “Martín-Estal, I., *et al.* (2022). Effect of Ethanol Consumption on the Placenta and Liver of Partially IGF-1-Deficient Mice: The Role of Metabolism via CYP2E1 and the Antioxidant Enzyme System. Biology, 11(9), 1264. https://doi.org/10.3390/biology11091264”.

- **The placental morphology should be analyzed more thoroughly with for example a quantification of the length ratio between JZ/labyrinth and/or the number of abnormal islets in the labyrinth (the quality of the image should also be improved as the text within the image is hardly readable). **

Thanks for the reviewer’s comments. Nevertheless, the quantification of the length ratio between JZ/labyrinth was performed, but no significant results were obtained (data not shown). The layout of images in Figure 2 was changed to make it more readable.

- **The AAH immunohistochemistry would also benefit for a quantification analysis (in specific layers). For all the quantitative data, the statistical analysis effectively used should be described with the indication of the number of analyzed samples per group. **

The reviewer is right. However, a quantification analysis of the immunohistochemistry images was  accomplished, but no significant results were obtained (data not shown).

- **Post hoc test should be performed after two-way ANOVA or Kruskal Wallis test, to compare the genotypes within the water or ethanol group, and compare the effect of ethanol within each genotype. Only with these additional analyses will it be possible to conclude more clearly on the results.**

After two-way ANOVA, two post-hoc tests were executed with SPSS software: Bonferroni and Tukey tests.

**More specific comments** **:**

1. **In Figure 3, there are Control: WT, n = 9; HZ, n = 32; KO, n = 22; Ethanol: WT, n = 23; HZ, n = 44; KO, n = 16. This does not fit with the expected ratio of 25/50/25% of WT/HZ/KO. It seems that there are some missing control WT. Could the authors explain this?**

Because of the treatment scheme being used in the present study, a greater number of females in the HZ-Ethanol group was used to decrease the risk of death or early delivery due to ethanol treatment.

  

1. **And for the RT-qPCR, it does not seem that all these placentas were analyzed. How were the one included chosen?**

To reduce variability between groups, only male fetuses were selected for RT-PCR analysis. In addition, the animal model could not be continued due Biotherium issues.

  

1. **Syncytiotrophoblast are in the labyrinth layer and not the junctional zone (see for example 10.3389/fimmu.2021.771054). The myometrium is not part of the placenta.**

Thanks for your comments. Trophoblast giant cells, the invasive cells responsible for invading maternal vasculature to promote placental development, are located in the labyrinth. However, spongiotrophoblast cells and glycogen trophoblast cells are found in the labyrinth and in the junctional zone (10.3390/nu15163564).

 Throphoblast invasion is observed in the junctional zone, in order to develop a normal placenta. Alterations in this trophoblast migration can lead to adverse pregnancy consequences due to a limit blood flow, such as fetal growth restriction and preeclampsia (10.1067/mob.2002.127305).

1. **For clarity, I would suggest to use Igf+/+, Igf +/- and Igf-/- rather than WT, HZ and KO throughout the manuscript.**

Thanks for the reviewer’s suggesting. The “Animals and experimental design” section explains the nomenclature used: WT ( *Igf1 ^+/+^*); HZ ( *Igf1 ^+/-^*) and KO ( *Igf1 ^-/-^*). We select that nomenclature because it was used in previous studies carried out by the research group with the present experimental model.

1. **The author should also discuss the fact that the decidua is always partially deficient in IGF1 which may influence placental establishment, as well as the disparity of mice age that could also influence the results.**

Although the decidua, which is in contact with the maternal side, is always partially deficient in IGF-1, the rest of the placenta depends on the fetus; that is, if the fetus is WT, the placenta will be of the same genotype. Also, IGF-2 has an important role in fetal growth due to its direct anabolic actions within the fetus, and by its modulation of placental supply capacity, which may be a compensatory mechanism for IGF-1 deficiency (10.1016/j.placenta.2018.01.005) (Lines: 385-388).

 On the other hand, the difference in age is not a large period, since it is a difference of 8 weeks, which is equivalent to 6.6 years in humans, a period in which the mice are still reproductively young (10.1016/j.lfs.2015.10.025).

1. **Did the ethanol consumption influence the weight gain of the mice throughout the study?**

Maternal parameters such as weight gain of pregnant dams, average of food and beverage consumption where measured throughout gestation. It was found that both HZ groups (HZ-Control and HZ-Ethanol) weighed less than the WT groups (WT-Control and WT-Ethanol), and this parameters was not affected by ethanol treatment. These results were reported in the following article: “Martín-Estal, I., *et al.*(2022). Effect of Ethanol Consumption on the Placenta and Liver of Partially IGF-1-Deficient Mice: The Role of Metabolism via CYP2E1 and the Antioxidant Enzyme System. Biology, 11(9), 1264. https://doi.org/10.3390/biology11091264”.

1. **How many HZ-ethanol female were actually mated?**

In HZ-Ethanol group, 9 female mice were mated with HZ male mice.

1. **The title should be: Ethanol consumption during gestation promotes placental alterations in IGF-1 deficient placentas.**

The reviewer is right. We will change the title as suggested, thanks.

  

1. **Is the fetal weight further reduced in Ethanol KO compared to water-KO?**

This result was reported in the following article: “Martín-Estal, I., *et al.* (2022). Effect of Ethanol Consumption on the Placenta and Liver of Partially IGF-1-Deficient Mice: The Role of Metabolism via CYP2E1 and the Antioxidant Enzyme System. Biology, 11(9), 1264. https://doi.org/10.3390/biology11091264”. In this article, the presence of KO genotype and ethanol consumption affected fetal height and weight when compared to the WT and HZ genotypes.

 Yours faithfully,

 Inma Castilla de Cortázar Larrea, MD, PhD.

 Professor of Medical Physiology

 HM Velazquez Cabinet

 Velazquez 25, 28001 Madrid, Spain

 Fabiola Castorena Torres, PhD.

 Research Professor, Coordinator of the Postgraduate Program in Biomedical Sciences

 Research and Innovation Department

 National School of Medicine and Health Sciences

 Tecnológico de Monterrey, México.
