# Supplementary material for: Ethanol consumption during gestation promotes placental alterations in IGF-1 deficient mice
Source: F1000Res. 2021 Dec 15;10:1284. [Version 1] doi: 10.12688/f1000research.75116.1 (PMC11617828; doi:10.12688/f1000research.75116.1)
Supplement: Peer review history

# Response

**Published:** 2024-03-04

**Authors:** Fabiola Castorena Torres

**Affiliations:** Tecnologico de Monterrey, Mexico

Monterrey, March 4 ^th^ 2024

 Dear Editorial Board members,

 First of all, I would like to thank you for your kind answer as for carefully revising the manuscript, that has greatly aimed us to improve our manuscript. Herein we will outline any changes made to the manuscript following reviewers’ advice (changes are shown in green in the manuscript) or, to the contrary, giving a rationale when a suggestion was not implemented.

**Reviewer 1:**

**Martín-Estal et al. performed a well-planned study to evaluate the effect of ethanol consumption on placental development (and fetal growth) in mice partially deficient for IGF1, as well as a potential synergy between (partial or total) IGF-1 deficiency and ethanol consumption on placental development. They notably showed that total IGF-1 deficiency and ethanol consumption both led to structural anomalies in the placental layers. They also showed a reduced placental efficiency in IGF-1 KO placentas, that was further reduced by ethanol consumption. They also evaluated the mRNA and protein expression of AAH and the mRNA expression of a few other genes.**

 We would like to thank the reviewer for his/her comments, which have helped us to improve the manuscript.

**While the data are interesting, their description and analysis is not sufficient.**

  

- **The fetal weight is an important element and thus should be presented. Fetal growth retardation/restriction is indeed a key aspect of IGF-1 signaling and ethanol consumption, as it is well described in the introduction. **

The reviewer is right. This information about fetal parameters (fetal weight, fetal height and placental weight) is presented in the following article of our research group: “Martín-Estal, I., *et al.* (2022). Effect of Ethanol Consumption on the Placenta and Liver of Partially IGF-1-Deficient Mice: The Role of Metabolism via CYP2E1 and the Antioxidant Enzyme System. Biology, 11(9), 1264. https://doi.org/10.3390/biology11091264”.

  

- **I would suggest adding 2 panels to Figure 3 with the fetal and placental weight (in addition to the ratio), this is especially important as the authors mentioned placental hypoplasia that is not visible in the presented data. **

The reviewer is right. Information is presented in the following article of our research group: “Martín-Estal, I., *et al.* (2022). Effect of Ethanol Consumption on the Placenta and Liver of Partially IGF-1-Deficient Mice: The Role of Metabolism via CYP2E1 and the Antioxidant Enzyme System. Biology, 11(9), 1264. https://doi.org/10.3390/biology11091264”.

  

- **The placental morphology should be analyzed more thoroughly with for example a quantification of the length ratio between JZ/labyrinth and/or the number of abnormal islets in the labyrinth (the quality of the image should also be improved as the text within the image is hardly readable). **

Thanks for the reviewer’s comments. Nevertheless, the quantification of the length ratio between JZ/labyrinth was performed, but no significant results were obtained (data not shown). The layout of images in Figure 2 was changed to make it more readable.

  

- **The AAH immunohistochemistry would also benefit for a quantification analysis (in specific layers). For all the quantitative data, the statistical analysis effectively used should be described with the indication of the number of analyzed samples per group. **

The reviewer is right. However, a quantification analysis of the immunohistochemistry images was  accomplished, but no significant results were obtained (data not shown).

  

- **Post hoc test should be performed after two-way ANOVA or Kruskal Wallis test, to compare the genotypes within the water or ethanol group, and compare the effect of ethanol within each genotype. Only with these additional analyses will it be possible to conclude more clearly on the results.**

After two-way ANOVA, two post-hoc tests were executed with SPSS software: Bonferroni and Tukey tests.

**More specific comments** **:**

1. **In Figure 3, there are Control: WT, n = 9; HZ, n = 32; KO, n = 22; Ethanol: WT, n = 23; HZ, n = 44; KO, n = 16. This does not fit with the expected ratio of 25/50/25% of WT/HZ/KO. It seems that there are some missing control WT. Could the authors explain this?**

Because of the treatment scheme being used in the present study, a greater number of females in the HZ-Ethanol group was used to decrease the risk of death or early delivery due to ethanol treatment.

  

1. **And for the RT-qPCR, it does not seem that all these placentas were analyzed. How were the one included chosen?**

To reduce variability between groups, only male fetuses were selected for RT-PCR analysis. In addition, the animal model could not be continued due Biotherium issues.

  

1. **Syncytiotrophoblast are in the labyrinth layer and not the junctional zone (see for example 10.3389/fimmu.2021.771054). The myometrium is not part of the placenta.**

Thanks for your comments. Trophoblast giant cells, the invasive cells responsible for invading maternal vasculature to promote placental development, are located in the labyrinth. However, spongiotrophoblast cells and glycogen trophoblast cells are found in the labyrinth and in the junctional zone (10.3390/nu15163564).

 Throphoblast invasion is observed in the junctional zone, in order to develop a normal placenta. Alterations in this trophoblast migration can lead to adverse pregnancy consequences due to a limit blood flow, such as fetal growth restriction and preeclampsia (10.1067/mob.2002.127305).

  

1. **For clarity, I would suggest to use Igf+/+, Igf +/- and Igf-/- rather than WT, HZ and KO throughout the manuscript.**

Thanks for the reviewer’s suggesting. The “Animals and experimental design” section explains the nomenclature used: WT ( *Igf1 ^+/+^*); HZ ( *Igf1 ^+/-^*) and KO ( *Igf1 ^-/-^*). We select that nomenclature because it was used in previous studies carried out by the research group with the present experimental model.

  

1. **The author should also discuss the fact that the decidua is always partially deficient in IGF1 which may influence placental establishment, as well as the disparity of mice age that could also influence the results.**

Although the decidua, which is in contact with the maternal side, is always partially deficient in IGF-1, the rest of the placenta depends on the fetus; that is, if the fetus is WT, the placenta will be of the same genotype. Also, IGF-2 has an important role in fetal growth due to its direct anabolic actions within the fetus, and by its modulation of placental supply capacity, which may be a compensatory mechanism for IGF-1 deficiency (10.1016/j.placenta.2018.01.005) (Lines: 385-388).

 On the other hand, the difference in age is not a large period, since it is a difference of 8 weeks, which is equivalent to 6.6 years in humans, a period in which the mice are still reproductively young (10.1016/j.lfs.2015.10.025).

  

1. **Did the ethanol consumption influence the weight gain of the mice throughout the study?**

Maternal parameters such as weight gain of pregnant dams, average of food and beverage consumption where measured throughout gestation. It was found that both HZ groups (HZ-Control and HZ-Ethanol) weighed less than the WT groups (WT-Control and WT-Ethanol), and this parameters was not affected by ethanol treatment. These results were reported in the following article: “Martín-Estal, I., *et al.*(2022). Effect of Ethanol Consumption on the Placenta and Liver of Partially IGF-1-Deficient Mice: The Role of Metabolism via CYP2E1 and the Antioxidant Enzyme System. Biology, 11(9), 1264. https://doi.org/10.3390/biology11091264”.

  

1. **How many HZ-ethanol female were actually mated?**

In HZ-Ethanol group, 9 female mice were mated with HZ male mice.

  

1. **The title should be: Ethanol consumption during gestation promotes placental alterations in IGF-1 deficient placentas.**

The reviewer is right. We will change the title as suggested, thanks.

  

1. **Is the fetal weight further reduced in Ethanol KO compared to water-KO?**

This result was reported in the following article: “Martín-Estal, I., *et al.* (2022). Effect of Ethanol Consumption on the Placenta and Liver of Partially IGF-1-Deficient Mice: The Role of Metabolism via CYP2E1 and the Antioxidant Enzyme System. Biology, 11(9), 1264. https://doi.org/10.3390/biology11091264”. In this article, the presence of KO genotype and ethanol consumption affected fetal height and weight when compared to the WT and HZ genotypes.

 Yours faithfully,

 Inma Castilla de Cortázar Larrea, MD, PhD.

 Professor of Medical Physiology

 HM Velazquez Cabinet

 Velazquez 25, 28001 Madrid, Spain

 Fabiola Castorena Torres, PhD.

 Research Professor, Coordinator of the Postgraduate Program in Biomedical Sciences

 Research and Innovation Department

 National School of Medicine and Health Sciences

 Tecnológico de Monterrey, México.
